# Identification of Distinct Characteristics of Antibiofilm Peptides and Prospection of Diverse Sources for Efficacious Sequences

**DOI:** 10.3389/fmicb.2021.783284

**Published:** 2022-02-04

**Authors:** Bipasa Bose, Taylor Downey, Anand K. Ramasubramanian, David C. Anastasiu

**Affiliations:** ^1^Department of Biomedical Engineering, San Jose State University, San Jose, CA, United States; ^2^Department of Computer Science and Engineering, Santa Clara University, Santa Clara, CA, United States; ^3^Department of Chemical and Materials Engineering, San Jose State University, San Jose, CA, United States

**Keywords:** antimicrobial, antibiofilm, machine learning, MBEC, MBIC, drug discovery

## Abstract

A majority of microbial infections are associated with biofilms. Targeting biofilms is considered an effective strategy to limit microbial virulence while minimizing the development of antibiotic resistance. Toward this need, antibiofilm peptides are an attractive arsenal since they are bestowed with properties orthogonal to small molecule drugs. In this work, we developed machine learning models to identify the distinguishing characteristics of known antibiofilm peptides, and to mine peptide databases from diverse habitats to classify new peptides with potential antibiofilm activities. Additionally, we used the reported minimum inhibitory/eradication concentration (MBIC/MBEC) of the antibiofilm peptides to create a regression model on top of the classification model to predict the effectiveness of new antibiofilm peptides. We used a positive dataset containing 242 antibiofilm peptides, and a negative dataset which, unlike previous datasets, contains peptides that are likely to promote biofilm formation. Our model achieved a classification accuracy greater than 98% and harmonic mean of precision-recall (F1) and Matthews correlation coefficient (MCC) scores greater than 0.90; the regression model achieved an MCC score greater than 0.81. We utilized our classification-regression pipeline to evaluate 135,015 peptides from diverse sources for potential antibiofilm activity, and we identified 185 candidates that are likely to be effective against preformed biofilms at micromolar concentrations. Structural analysis of the top 37 hits revealed a larger distribution of helices and coils than sheets, and common functional motifs. Sequence alignment of these hits with known antibiofilm peptides revealed that, while some of the hits showed relatively high sequence similarity with known peptides, some others did not indicate the presence of antibiofilm activity in novel sources or sequences. Further, some of the hits had previously recognized therapeutic properties or host defense traits suggestive of drug repurposing applications. Taken together, this work demonstrates a new *in silico* approach to predicting antibiofilm efficacy, and identifies promising new candidates for biofilm eradication.

## 1. Introduction

Many microbes in their natural habitats are found not as free-floating (planktonic) organisms, but as three dimensional aggregates encased in a polymeric matrix called biofilms (Costerton et al., 1987). Biofilms are responsible for 65–80% of recalcitrant infections in humans. Once established, biofilms have the potential to initiate or prolong infections by providing a safe sanctuary from which organisms can invade local tissue, seed new infection sites and resist eradication efforts. Both bacteria and fungi form biofilms on abiotic (e.g., catheters and implants) or biotic (e.g., skin, wounds) surfaces (Torres et al., 2018; Ramasubramanian and Lopez-Ribot, 2019). Cells within the biofilms display high levels of resistance against clinically-administered antibiotics, which often leads to morbidity and mortality (Srinivasan et al., 2017). Therefore, there is an urgent need to develop agents that are effective against biofilm infections (de la Fuente-Núñez et al., 2013; Pierce et al., 2015).

The traditional antibiotic screening paradigm first established during the “golden era” of antibiotics (1940–1960), which has continued until very recently, was heavily biased toward the discovery of “magic bullets” that have either bacteriostatic or bactericidal properties but elicited waves of antibiotic resistance. This approach has had little success against multidrug resistant (MDR) highly virulent *Enterococcus faecium, Staphylococcus aureus, Klebsiella pneumoniae, Acinetobacter baumannii, Pseudomonas aeruginosa*, and *Enterobacter spp* (ESKAPE) pathogens (De Oliveira et al., 2020). Antimicrobial peptides (AMP) have emerged as a promising alternative or complement to chemical compounds in treating microbial infections (Margit et al., 2016). More than 4,700 such peptides have been identified in all forms of life, and are deposited in the Antimicrobial peptide database (APD) (Wang et al., 2015). Compared to chemical antibiotics, AMPs are particularly attractive for several reasons: (i) AMPs appear to have a lower rate of inducing bacterial resistance and they continue to be developed clinically (Spohn et al., 2019); (ii) AMPs appear to be the last resort for recalcitrant infections as exemplified by Polymyxin B, colistin, daptomycin against the MDR ESKAPE pathogens (Zavascki et al., 2007); (iii) AMPs can work synergistically with antibiotics (Sheard et al., 2019).

In the recent past, several Machine Learning (ML)-based approaches have been developed for the characterization and prediction of novel AMPs including AntiBP - for predicting antibacterial peptides (Lata et al., 2007), iAMP-2L-for identifying antimicrobial peptides (Xiao et al., 2013), iAMPred-for predicting antimicrobial peptides by using physico-chemical and structural properties (Meher et al., 2017), AmPEP-for sequence based prediction of antimicrobial peptides (Bhadra et al., 2018). These studies have clearly demonstrated that pattern-based computational approaches to establish structure-function relationships are a powerful alternative or augmentation to experimental biochemical assays, which are inherently lower throughput, and expensive. More importantly, ML approaches have been used to discover new AMP sequences (Lee et al., 2016), predict unknown peptides from known ones (Fjell et al., 2008), identify peptides with multiple functions (Haney et al., 2018), and to discover previously unknown interrelationships between existing peptides (Lee et al., 2016).

While a vast majority of work has focused on AMPs effective against microbial infections in general, relatively fewer experimental or computational efforts have been invested on discovering peptides that are effective against biofilm infections. These peptides, called Antibiofilm peptides (ABP) are a subset of AMPs that inhibit biofilm formation or eradicate previously formed biofilms. Nearly 200–300 peptides have been identified to be effective against biofilms and are listed in the antibiofilm peptide database, BaAMPs (Luca et al., 2015). ABPs can be particularly attractive as a strategy to limit microbial virulence without necessarily killing the organisms, or risking the development of antibiotic resistance. ABPs can be used as an alternative to antibiotics in microbial infections (Pletzer and Hancock, 2016).

Previous ML approaches have focused on establishing patterns from existing antibiofilm peptides that enable the classification of candidate peptides for potential antibiofilm activity (Gupta et al., 2016; Sharma et al., 2016; Fallah Atanaki et al., 2020). Gupta et al. developed sequence-based support vector machine (SVM) and random forest (RF) models to predict antibiofilm activity using the peptides listed in the BaAMP database. Their model achieved reasonable success with a Matthews's correlation coefficient (MCC) score of 0.84 (Gupta et al., 2016). Sharma et al. developed SVM- and Weka-based models using BaAMP data as their positive dataset, and quorum-sensing peptides as their negative set. They achieved an MCC of 0.91 (Sharma et al., 2016). Another web-based model, BIPEP, was developed by Fallah Atanaki et al. (2020) wherein peptides from the APD and BaAMP databases were used as the positive set, along with a negative dataset consisting fewer quorum sensing peptides that in the positive set. Their SVM model achieved an MCC value of 0.89. While these studies developed important quantitative structure and activity relationships in ABPs, they suffered from some drawbacks which may affect the model performance. The model of Atankai et al. did not account for the lower abundance of ABPs in nature. The model of Gupta et al. might have used the pattern recognition sequences (“motifs”) as a privileged information prior in the classification model, while the model of Sharma et al. considered a smaller negative set compared to their positive data set. Most importantly, these models can only classify ABPs but do not provide any insights into the efficacy of these peptides against biofilms.

The objectives of this work are 3-fold: first, we seek to improve the classification algorithm for ABPs by using a more realistic, curated negative dataset with mostly biofilm-favoring peptides which is 10-fold larger than the positive dataset; our model identifies the most useful amino-acid composition features and short-repeating patterns (“motifs”) indicative of antibiofilm activity; second, we seek to develop a regression model using the minimum biofilm inhibitory concentration (MBIC) and minimum biofilm eradication concentration (MBEC) of ABPs to predict the effectiveness of the novel peptides classified as antibiofilm candidates; third, we seek to understand the putative mechanisms of action of the peptide hits using their previously known properties, secondary structure, and similarity with known antibiofilm peptides.

## 2. Methods

### 2.1. Dataset Preparation

In this work, we collected data with the aim to improve the performance of antibiofilm prediction models. Since biofilm eradication is a well-defined physiological phenomenon, any peptide will have less than a random 50% chance to be active against preformed biofilms. Therefore, instead of using a balanced dataset consisting of equal amounts of ABP and non-ABP, we used an imbalanced, operationally tractable dataset consisting of ten times more non-ABP peptides in the negative dataset compared to ABP peptides in the positive dataset. We chose to work with real peptides instead of randomly generated peptides so that a more realistic performance may be obtained from our classifier models. Therefore, we collected peptides which directly or indirectly could play a role in biofilm formation as elaborated in section 2.1.1. For establishing the efficacy, we performed an extensive literature search to obtain peptides with minimum biofilm inhibitory concentration (MBIC), and minimum biofilm eradication concentration (MBEC).

#### 2.1.1. Dataset 1

We extracted ABPs from the Antimicrobial Peptide Database (APD), and the Biofilm-active Antimicrobial Peptide database (BaAMP). After removing duplicates, we obtained 242 ABPs, which served as our positive dataset (Supplementary Tables S14–S18 in Supplementary Note 6). For the negative dataset, we curated peptides from different databases such as UniProt (Consortium, 2020), Quorum Sensing Peptide Prediction Server (QSPProd) (Rajput et al., 2015) and NCBI protein database (Coordinators, 2016). The peptides from the UniProt database were screened for their direct or indirect contribution to biofilm formation, including regulation, association with biofilm matrix polysaccharide or proteins, and association with the cells themselves. For example, we added protein Q59U10, which is a biofilm and cell wall regulator in *Candida albicans*. We also screened the proteomic profiles of different biofilm-forming bacteria like *Staphylococcus aureus* and *Escherichia coli*, and included peptides from the NCBI and UniProt databases that promote biofilm formation. For example, we included fibronectin-binding protein B, which promotes the accumulation and surface attachment of biofilm by *Staphylococcus aureus*. We also included quorum sensing peptides, which promote biofilm formation, from QSPProd in our negative dataset. To have a sequence length distribution in the negative dataset that is similar to the positive dataset, we considered either only the signal peptide length of the original protein, or we divided the whole sequence into several sequences of length 70–75, depending on the protein length. One caveat with this approach is that fragments from a biofilm-promoting protein may not retain the property of the parent protein. The negative dataset has peptides of length 4–75.

Eighty percent of the positive and negative datasets were used for training and 10-fold cross-validation while the remaining 20% was kept aside as a test/validation set. The performances of different machine learning algorithms were evaluated on this out-of-scope test dataset.

#### 2.1.2. Dataset 2

We curated the minimum biofilm inhibitory concentration (MBIC), and the minimum biofilm eradication concentration (MBEC) for our positive dataset against different gram-positive and gram-negative bacteria from the source publications. In cases where these values were not listed in the source publication, the approximate values were obtained from images or graphs in the respective articles. For example, for LL-37, we consider the case where *P. aeruginosa* biofilm were grown previously and then peptides were added in various concentration (Nagant et al., 2012). The bacteria was tagged with green fluorescent protein and the killed biofilm appeared as red in the result. We analyzed the figures, which indicate that the killing starts at 20 μM concentration. Therefore, we considered 20 μM as the MBEC value of LL-37 against *P. aeruginosa*. Likewise we did the search of all the ABPs from our positive dataset. Of the 242 peptides in our positive dataset, we obtained MBIC and MBEC values for 178 and 57 peptides (Supplementary Tables S19, S20 in Supplementary Note 4), respectively.

We did not consider the peptides which showed inhibition/eradication against fungal pathogens like *Candida* and others.

#### 2.1.3. Candidate Dataset

In addition to the labeled dataset we used to train and evaluate the performance of our computational antibiofilm prediction models, we constructed a large *candidate dataset* from various sources, including 74 anticancer, 220 antiviral, and more than 4,770 antimicrobial peptides from the Data Repository of AntiMicrobial peptides (DRAM) (Kang et al., 2019). Additionally, we collected all 202,716 peptides from UniProt of sequence length 11–20. After removing duplicates, our candidate dataset contains 109,807 unique UniProt peptides. We also included peptides from the Swiss-Prot section of UniProt (Duvaud et al., 2021) with sequence length 4–10 and 20–80. In total, we tested our model against 135,015 unique peptides from different data sources.

### 2.2. Feature Extraction

We used the “propy3” (Cao, 2020) and the “protParam” (Cock et al., 2009) software packages to extract different peptide features, which are numerical representations of the peptide sequence, structure, and physicochemical properties as described below.

#### 2.2.1. Amino Acid Composition

The Amino Acid Composition (AAC) features represent the percentage of each amino acid present in the peptide sequence. The biopython package returns a 20-element vector of the naturally occurring amino acids. Equation 1 provides the formula for computing the AAC of a given amino acid *i*:


(1) $$\begin{array}{l} AAC\left(i\right)=\frac{\#\;amino\;acids\;of\;type\;\left(i\right)}{\#\;amino\;acids}\times 100 \end{array}$$

#### 2.2.2. Dipeptide Composition

Dipeptide Composition (DPC) represents the percentage of the dipeptides present in the peptide sequence. The DPC feature returns 400 named vectors with a non-zero value for any amino acid pair (dipeptide) present in the peptide.


(2) $$\begin{array}{l} DPC\left(i,j\right)\;=\frac{\#\;dipeptide\;\left(i\;and\;j\right)}{\#\;possible\;dipeptides}\times 100 \end{array}$$

#### 2.2.3. Composition, Transition, Distribution

The Composition, Transition, Distribution (CTD) descriptor is a 147-element vector representing different physio-chemical properties of the peptides (Xiao et al., 2015). The properties of peptides that are part of the CTD descriptor include “hydrophobicity,” “normalized van der Waals volume,” “polarity,” “polarizability,” “charge,” “secondary structure,” and “solvent accessibility.” The amino acids are divided into three groups depending on their property and functionality. The “composition” features represent the percentage of each group of amino acids in the peptide. The “transition” features represent the relative frequencies of a given amino acid from one group being followed by an amino acid from a different group. Finally, the “distribution” features represent the percentage residue of each attribute present in the peptide in their first, 25%, 50%, 75%, and 100% of residues, respectively.

#### 2.2.4. Motif

“Motifs” are maximal length amino acid sequences present in peptides which may represent a unique biological or chemical function. We used the “MERCI” software (Vens et al., 2011) to identify distinct patterns in ABPs that are not present in non ABPs (non-antibiofilm peptides). The MERCI software provides two scripts to extract motifs. One script can essentially find all the motifs that are present in the positive dataset and absent in the negative dataset, which was used to discover and store, in each experiment, motifs found in our training dataset. We then used the second script to identify which of those training set motifs were present in the test samples. Finally, we used the number of identified motifs found in a given peptide as the motif-based single-variate feature.

#### 2.2.5. Other Features

We extracted other critical, global features, such as sequence length, molecular weight, aromaticity, and isoelectric point, using the “ProteinAnalysis” module of the “protParam” software.

### 2.3. Machine Learning Models

We developed our prediction model using several machine learning algorithms, including Support Vector Machines (SVM), Random Forest (RF), and Extreme Gradient Boosting (XGBoost) classifiers. Our goal was to select the algorithm that provides the best predictive performance for antibiofilm activity on out-of-sample data. We used the “Scikit-learn” (Pedregosa, 2011) package to train and test models for our work.

#### 2.3.1. Support Vector Machine

Support Vector Machine (SVM) is one of the most commonly used classifiers for peptide prediction (Ng et al., 2015). SVM works particularly well for binary classification problems. The model works by separating samples in different classes using a hyperplane, which can be expressed in a high dimensional space through kernel transformations. Since our dataset is not relatively large, we used a nonparametric method that SVM supports and a radial basis function (RBF) kernel. SVM is a robust model that can be used for both classification and regression. Literature shows that SVM has performed exceptionally well in predicting peptide function (Gupta et al., 2013).

#### 2.3.2. Support Vector Regressor

The Support Vector Regressor (SVR) model uses the same principle as SVM, but for regression problems. Instead of separating samples into different classes using a hyperplane, the hyperplane is used to create a best fit line that has the maximum number of points between the decision boundaries. Like the classifications models, we used a radial basis function (RBF) kernel to create a nonlinear hyperplane. The SVR was used to predict minimum biofilm eradication/inhibitory concentration (MBEC/MBIC).

#### 2.3.3. Random Forest

The Random Forest (RF) model is an ensemble prediction model, which also supports both regression and classification. RF has been used to classify peptides and to solve other biological problems (Manavalan et al., 2018). Although RF may not be the best choice as a classifier for an imbalanced dataset, we used this algorithm to compare the performance with other classification algorithms.

#### 2.3.4. Extreme Gradient Boosting

The Extreme Gradient Boosting (XGBoost) model is comparatively a new prediction method used in machine learning, which can also be used for both classification and regression problems. In our work we used the XGBClassifier. The XGBoost algorithm has regularization parameters that can be tuned to reduce overfitting in an imbalanced dataset. This algorithm is also used in prior work for the prediction of peptides with an accuracy greater than 98% (Wang et al., 2020).

### 2.4. Cross-Validation and Stratified Sampling

To address potential overfitting problems, we performed 10-fold cross-validation of our training dataset, wherein one part of the dataset, called the validation set, was used for testing, and the other nine were used for training. This process was iterated over ten times, using, in turn, each of the ten parts as the validation set. Since our dataset is imbalanced, having ten negative peptides for every positive one, we used stratified sampling to ensure that each fold receives an equal percentage of positive and negative peptides while doing cross-validation. Additionally, we used stratified sampling to ensure that the out-of-sample test dataset also has precisely 20% of the positive data, i.e., 48 peptides, and 20% of the negative data, i.e., 485 peptides. The details of the distribution of dataset is available in Supplementary Table S1 in Supplementary Note 1.

### 2.5. Performance Evaluation

We used several standard metrics to evaluate our models” performance, including sensitivity (Sen), specificity (Spec), accuracy (Acc), Matthews's correlation coefficient (MCC), and harmonic mean of the precision-recall (F1) Score. The metrics are defined as,


(3) $$\begin{array}{l} Specificity=\frac{TN}{FP\;+\;TN} \end{array}$$

(4) $$\begin{array}{l} Sensitivity=\frac{TP}{TP\;+\;FN} \end{array}$$

(5) $$\begin{array}{l} Accuracy=\frac{TP\;+\;TN}{TP\;+\;FP\;+\;TN\;+\;FN} \end{array}$$

(6) $$\begin{array}{l} F1=\frac{TP}{TP\;+\;\frac{FP\;+\;FN}{2}} \end{array}$$

(7) $$\begin{array}{l} MCC=\frac{\left(TP\right)\left(TN\right)\;-\;\left(FP\right)\left(FN\right)}{\sqrt{\left(TP+FP\right)\;\left(TP+FN\right)\;\left(TN+FP\right)\;\left(TN+FN\right)}} \end{array}$$

where, TP = True Positive, TN = True Negative, FP = False Positive, and FN = False Negative. For each model we tested, we used 10-fold cross-validation to tune meta-parameters and find the best model performance on the training set. We report the effectiveness of that model on the out-of-sample test set in the following section.

### 2.6. Principal Component Analysis (PCA)

During feature selection, the samples were transformed into a lower dimensional space via Principal Component Analysis (PCA). Several hyperparameters were tuned, namely the regularization parameter (C) and kernel coefficient (γ) for the SVM/SVR models, and the number of principal components for the dimensionality reduction. We employed 5-fold stratified cross validation for classification and 5-fold cross validation for regression to ensure we trained a generic enough model that would not overfit the training set.

### 2.7. Sequence Alignment and Structure Prediction

Sequence alignment of peptides was performed to identify structural similarities between peptides using Clustal Omega, and visualized using Jalview (Madeira et al., 2019). The BLOSUM62 raw scores were used to confirm pairwise homology. To obtain consensus sequences from multiple sequence alignments, the first phylogenetic relationship was established based on BLOSUM62 scores. Then, from peptides in closely related trees, same or highly similar residues were extracted. To obtain the degree of disorder, the DisEMBL algorithm was used, and sequences with a threshold value greater than 0.55 were classified as “hotloops” or highly disordered regions. The 2D and 3D structures of the peptides were predicted using the PEP2D (Singh et al., 2019) and PEP-FOLD3 (Lamiable et al., 2016) servers, respectively.

## 3. Results and Discussion

**Figure 1:**
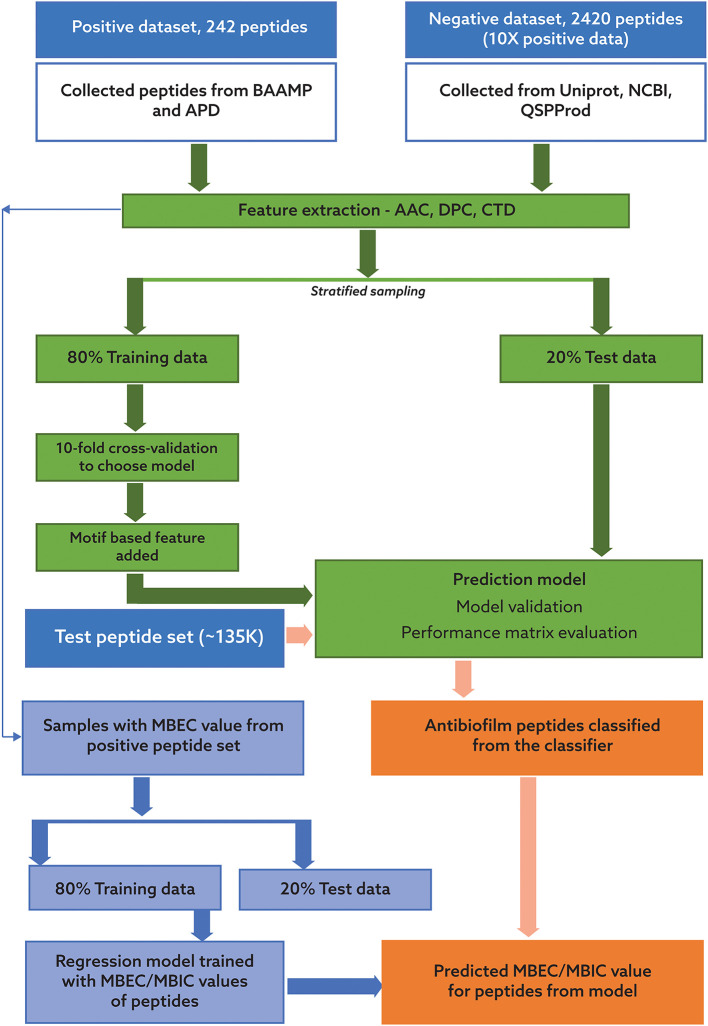
Process flow for the classification of potential antibiofilm peptides and prediction of antibiofilm activity. The process consists of two distinct, sequential steps. In the first step, a binary classification model was trained using a dataset with 242 peptides with reported antibiofilm activity and 2420 peptides with no known or suspected antibiofilm activity. In the second step, two regression models were trained using a subset of the peptides with known minimum biofilm eradication concentration (MBEC) and minimum biofilm inhibitory concentration (MBIC) values. Candidate peptides will be first evaluated for potential antibiofilm activity using the classification model, and then their effectiveness will be predicted using the regression model.

Our pipeline to predict peptides active against biofilms may be grouped into four key steps: identification of positive and negative datasets; development of a robust machine learning algorithm for classification of ABPs; collection of candidate potential ABPs from diverse habitats; and prediction of the efficacy of the novel peptides using our antibiofilm peptide classification model and a regression model based on known MBEC data. In the following, we will describe each of these tasks, which are also portrayed in Figure 1.

### 3.1. Characteristics of Peptides in the Positive Dataset

#### 3.1.1. Sequence Length

**Figure 2:**
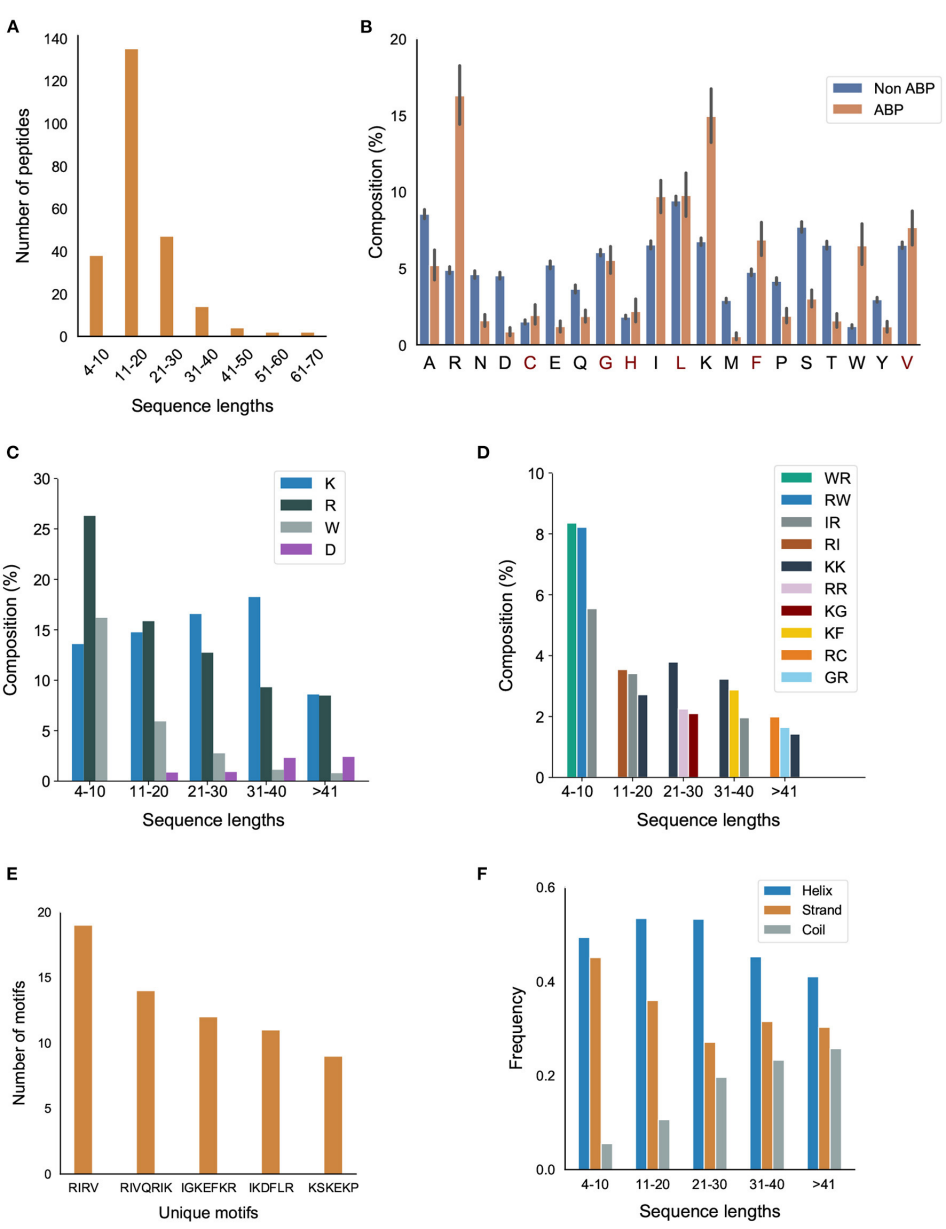
Primary and secondary structural characteristics of antibiofilm peptides. **(A)** Distribution of antibiofilm peptides over different sequence lengths; **(B)** Comparison of amino acid distribution in the positive and negative datasets. Statistical significance (*p* ≤ 0.001) was established using Mann-Whitney U-test; Amino acids marked in “red” on the x-axis of the figure are not statistically significant; **(C)** Distribution of K, R, and W, grouped based on the peptide length; **(D)** Dipeptide composition analysis shows IR, RI, WR, RW, and KK are the most common dipeptide sequences present in the antibiofilm peptide database (AA, LL, AL, or LA are most common in the negative dataset); **(E)** Most commonly found motifs in the antibiofilm peptides; **(F)** Antibiofilm peptides contain more alpha helices than beta sheets or coils.

The number of amino acids in our positive dataset varies between 4–70 (Figure 2A). Almost all the peptides have a sequence length less than 50. Only 2 peptides have a sequence length between 50–60 and 2 peptides have a sequence length between 60–70. Most of the ABPs were relatively short, i.e., two-thirds of the peptides contain less 20 amino acids with half of the peptides containing between 11–20 amino acids.

#### 3.1.2. Amino Acid Composition

We compared the distribution of 20 amino acids in the peptides in the positive and negative datasets (Figure 2B). We observed that, compared to the negative dataset, the ABPs contained a significantly higher percentage of lysine (K), arginine (R), tryptophan (W), and a significantly lower percentage of aspartic acid (D), glutamic acid (E), threonine (T) serine (S), asparagine (N), and methionine (M). This clearly indicates that the ABPs are positively charged, and contain a lower fraction of polar but uncharged side chains. The higher percentage of W indicates a higher hydrophobic nature of ABPs. This is further exemplified when the distribution of amino acids in the positive dataset were grouped by sequence length: K, R, and W make up 50% of the amino acid composition in the short peptides (<20 amino acids), which make up two-thirds of the positive dataset (Figure 2C). We also observed that peptides contain non-polar amino acids isoleucine (I), leucine (L), glycine (G), and alanine (A), which provide an amphipathic character to the ABPs.

#### 3.1.3. Dipeptide Composition

Figure 2D and Supplementary Figure S1 in Supplementary Note 2 show the most commonly encountered dipeptides in our positive and negative datasets, respectively. In our analysis, we focused on the top 3 dipeptide components in different sequence ranges. With the higher prevalence of K, R, and W in the ABPs, all the top candidates for dipeptides contained these amino acids. When arranged by sequence length, it can be seen that the majority of the peptides (>80%, with lengths in the range 4–30) contained “WR/RW,” “RI/IR,” “KK,” and “RR” as the most commonly encountered dipeptides. In contrast, the non-ABPs have non-polar aliphatic amino acid leucine (L) and alanine (A) in the top 5 dipeptide candidates. The dipeptide components most prominent in the positive and negative datasets clearly indicate the presence of a higher percentage of cationic and hydrophobic amino acids, and charge-hydrophobicity as a recurring theme in the antibiofilm (positive) dataset. It is this recurring presence of the charge-hydrophobicity combination exemplified by the dipeptide composition that underscores the amphipathic characteristic typically attributed to the action of ABPs.

#### 3.1.4. Motifs

Motifs represent short sequences that are commonly found in the datasets. We observed that the motifs “RIRV,” “RIVQRIK,” and “IGKEFKR” appeared more frequently in the positive dataset (Figure 2E), indicating a combination of polar and non-polar amino acids as one of the main reasons of amphipathicity. In contrast, the most prevalent motifs in non-ABPs were “SE,” “ET,” and “VD,” mainly consisting of acidic amino acids, i.e., aspartic acids and glutamic acids. This analysis shows that certain motifs, although present in a relatively smaller fraction, can be effective in conferring antibiofilm properties. For instance, human cathelicidin LL-37 prevents biofilm formation of *P.aeruginosa* at a concentration lower than its MIC value by probably blocking the growth of the extracellular matrix (Nagant et al., 2012). While the truncated LL19-37 did not affect biofilm growth, the addition of “IGKEFK” (LL13-37) inhibited biofilm formation at 50 μM. “IGKEFK” is one of the motifs we found in high numbers in our positive dataset during motif analysis. As stated in Nagant et al. (2012) LL-19 has no activity against bacterial membrane permeability, but adding a motif of “IVQRIK” increases permeability in LL-25. “IVQRIK” is another motif that we found in our positive dataset.

#### 3.1.5. Secondary Structure Analysis

We observed that ABPs of any sequence length are more likely to form α-helix structures (Figure 2F). In smaller length peptides, we noticed a prevalence of α-helix structures. As the peptide length increases, we noticed a greater percentage of coils in the peptides. The presence of positively charged and hydrophobic amino acids, together with the propensity to form α-helices suggests that the predominant mechanism of antibiofilm activity consists of positively charged, amphipathic helical peptides.

#### 3.1.6. Physicochemical Properties

**Figure 3:**
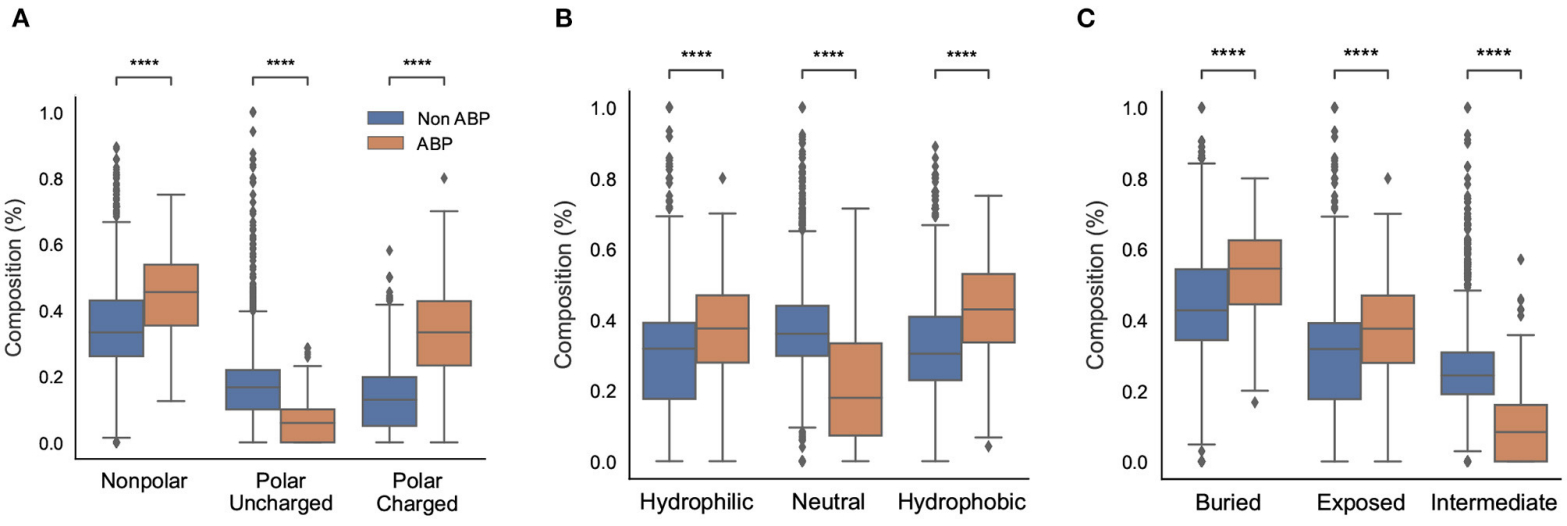
Physicochemical characteristics of antibiofilm peptides. **(A)** Polarity; **(B)** Hydrophobicity; **(C)** Solvent accessibility. Statistical significance (****p ≤ 0.001) was established using the Mann-Whitney *U*-test using python package.

We also compared different physicochemical properties, namely, polarity, hydrophobicity, and solvent accessibility between the positive and negative datasets (Figure 3). First, as expected from the AAC, the ABPs, compared to the negative dataset, contained a significantly larger fraction of charged polar residues but a smaller fraction of uncharged polar residues. Second, the comparison of hydrophobic properties between ABPs and non-ABPs showed that ABPs in our dataset consisted of a much lower number of hydropathically neutral peptides but significantly higher number of hydrophobic or charged residues. The hydrophobic portion of ABPs leads to insertion of the peptides into the less polar bacterial membrane and to destabilizing membrane barriers (Schmidt and Wong, 2013). The higher percentage of alanine, valine, leucine, isoleucine, and phenylalanine could be a potential reason for the ABP's hydrophobic nature. Third, ABPs, compared to the negative dataset, had a significantly lower percentage of compounds that were neutral in their interactions with solvent water but contained more residues that will be buried or exposed when exposed to water. This compositional analysis shows that the ABPs are composed of amino acids with strongly polar and hydrophobic tendencies which together provide an amphipathic nature rather than neutral amino acids.

### 3.2. Performance of Machine Learning Models

#### 3.2.1. Classifier Performance

**Figure 4:**
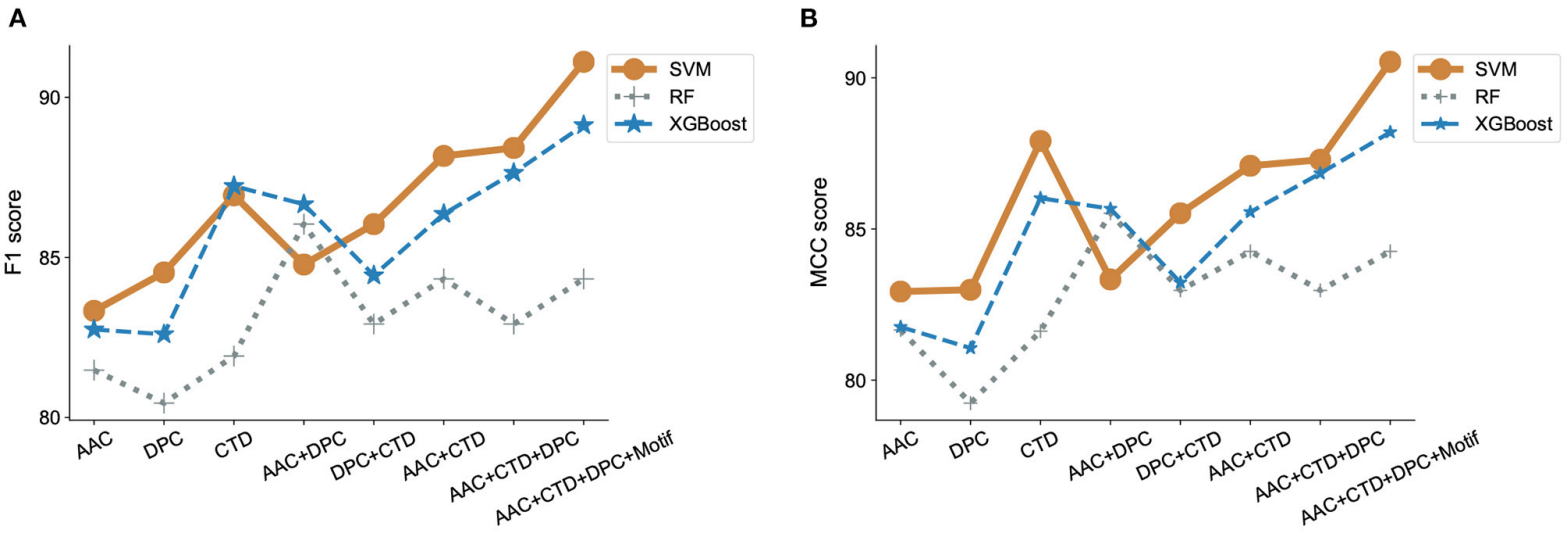
Evaluation of classification models and features. F1 scores **(A)** and MCC **(B)** were estimated for three different machine learning algorithms, SVM, RandomForest and XGBoost, incorporating features that contain combinations of amino acid composition (AAC), dipeptide composition (DPC), composition-transition-distribution parameters (CTD), and motifs. SVM performed best when run against all features, including motif.

Having characterized the antibiofilm peptide dataset, we used primary and secondary structure information of the peptides to develop machine learning models to identify and understand features that may be unique to ABPs. We used a total of 572 features obtained from AAC, DPC, and CTD analysis, and motifs, in various combinations, to describe our peptide samples numerically and train our machine learning models. For the SVM model, we used a linear kernel with and without *recursive feature elimination*, and a RBF kernel. Recursive feature elimination is a heuristic method used to select a subset of the features that may lead to superior performance compared to using all initial features; it works by iteratively eliminating the feature whose elimination produces the most improvement in performance, until no such performance improvement can be achieved. The linear kernel in SVM did not perform well and only achieved an MCC score less than 0.75. Additionally, recursive feature elimination did not lead to improvements in our classification model performance. Using the radial bias kernel provided the highest model performance. For the XGBoost and Random Forest models, the model parameters, number of estimators and maximum depth, were tuned. The performance of the models are presented in Figure 4 and Supplementary Tables S2–S4 in Supplementary Note 3. The accuracy for all the models was more than 95% while the model specificity varies between 98 and 100%. Since our model is a binary classifier and our dataset is not balanced, we used F1 score and MCC as the two key metrics to evaluate the performance of these machine learning models (Figure 4).

We observed that using either AAC, DPC, or CTD alone resulted in an F1-score between 0.80 and 0.85 in all our models. In contrast, using a combination of two of the three sets of features significantly improved the model performance with F1-scores between 0.82 and 0.88 in all our models. Using a combination of all three sets of features further, though modestly, improved model performance. We also observed that the addition of motifs as a feature improved the performance of our classifier model with SVM (Figure 4A). Similar observations have been noticed with MCC scores. While only considering DPC gave an MCC as low as 0.79, adding the remaining features lead to an MCC score of 0.9 (Figure 4B).

To adequately compare the performance of our model with that of previously published models on ABPs, we applied our best-performing model to the dataset used by Gupta et al. (2016). It is important to mention here that the performance obtained by one model vs. another on the same task cannot be directly compared unless experiments were conducted using the same dataset and evaluation metrics. Our best performing model is SVM with a radial bias kernel that utilizes AAC, DPC, CTD, and motif as features. Our model outperformed the previously reported results by a significant margin as seen by the increase in MCC from the published value of 0.84 in Gupta et al. (2016) to 0.90 (Supplementary Table S5 in Supplementary Note 3).

#### 3.2.2. Distinguishing Characteristics of Antibiofilm Peptides Predicted by the SVM Model

Next, we ranked features that are distinct in the positive dataset compared to the negative dataset, so that we may be able to ferret out higher level information that may be unique about the ABPs. To this end, the features were ranked in the order of increasing importance, as determined by the SVM with radial RBF kernel model.

There is no available API in the *scikit learn* RBF kernel SVM package to get the top feature. Therefore, we used a forward selection method to choose the top features. This is a computationally intensive iterative process where we start with zero features and iteratively add each feature that leads to the most performance improvement, until all 572 features are exhausted. In essence, it is the opposite of the recursive feature elimination method. With each iteration, the algorithm identifies the next best performing feature. The performance score was measured using the MCC score.

**Figure 5:**
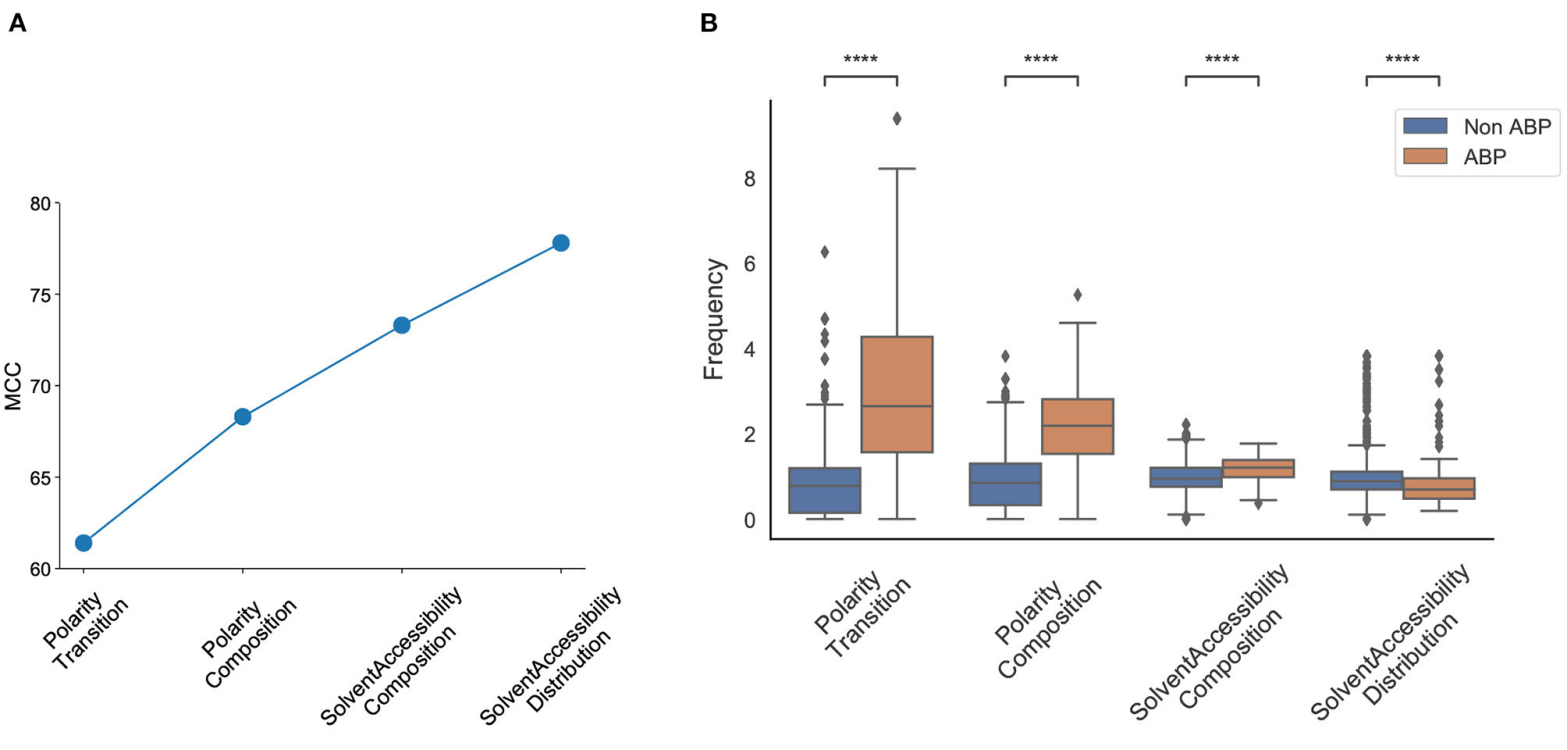
Most discriminant features obtained from the SVM model using Forward Selection. **(A)** Cumulative increase in MCC with top features shows that polarity and solvent accessibility are sufficient to account for nearly all the differences between the positive and negative datasets; **(B)** Distribution of top features between the positive and negative datasets. Polarity Transition means transition from group1 (non-charged) to group3 (highly charged) amino acids; Polarity Composition means composition of highly charged amino acids; Solvent Accessibility Composition means composition of buried amino acids; Solvent Accessibility Distribution means the fraction of first 25% of residues in the sequence are buried amino acids. Statistical significance (*p* ≤ 0.001) was established using the Mann-Whitney U-test from python package.

We observed that the first feature generated an MCC score of 0.61, and the first four features generated an MCC score of 0.79. The addition of the next four and eight features generated an MCC score of 0.81, and 0.82, respectively; the inclusion of all 572 features generated an MCC score of 0.91 (Figure 5A). The first four features that contributed most to the MCC score were all associated with the physicochemical properties of the peptides, namely, number of transitions from apolar to polar amino acids, fraction of polar amino acids, fraction of amino acids that are buried and least accessible to solvent, and distribution of solvent accessibility for amino acids that are buried from first residue to 25% residue. The distributions of these parameters in the positive and negative datasets reveal non-overlapping distributions, which further explains their performance impact in the classification task (Figure 5B). This analysis confirmed the importance of the alternation between charge and hydrophobicity. The most discerning feature from the forward selection process, “polarity-transition-group-1-3,” is the transition from polar group 1, i.e., from non-polar sequences like Gly, Ala, Val, Leu, Ilu, Pro to group 3, i.e., charged-polar amino acids like His, Lys, and Arg. The other discerning features consist of “polarity-composition-group-3” or composition of highly charged amino acids like His, Lys and Arg, “Solvent-Accessibility-composition-group-1” or composition of buried amino acids, and “Solvent-Accessibility-Distribution-group1” or distribution of amino acids like Ala, Leu, etc.

#### 3.2.3. Prediction of Antibiofilm Efficacy Using Regression Models

The effectiveness of ABPs are evaluated based on their minimum biofilm inhibitory concentration (MBIC) and minimum biofilm eradication concentration (MBEC) levels. The MBIC represents the concentration of the peptide that will prevent biofilm formation, while MBEC represents the concentration of the peptide that can remove preformed biofilms. datasets containing both concentrations were modeled and evaluated for efficacy, with the goal of predicting these values for antibiofilm peptide hits.

##### 3.2.3.1. Models for the Prediction of Minimum Biofilm Inhibitory Concentration

**Figure 6:**
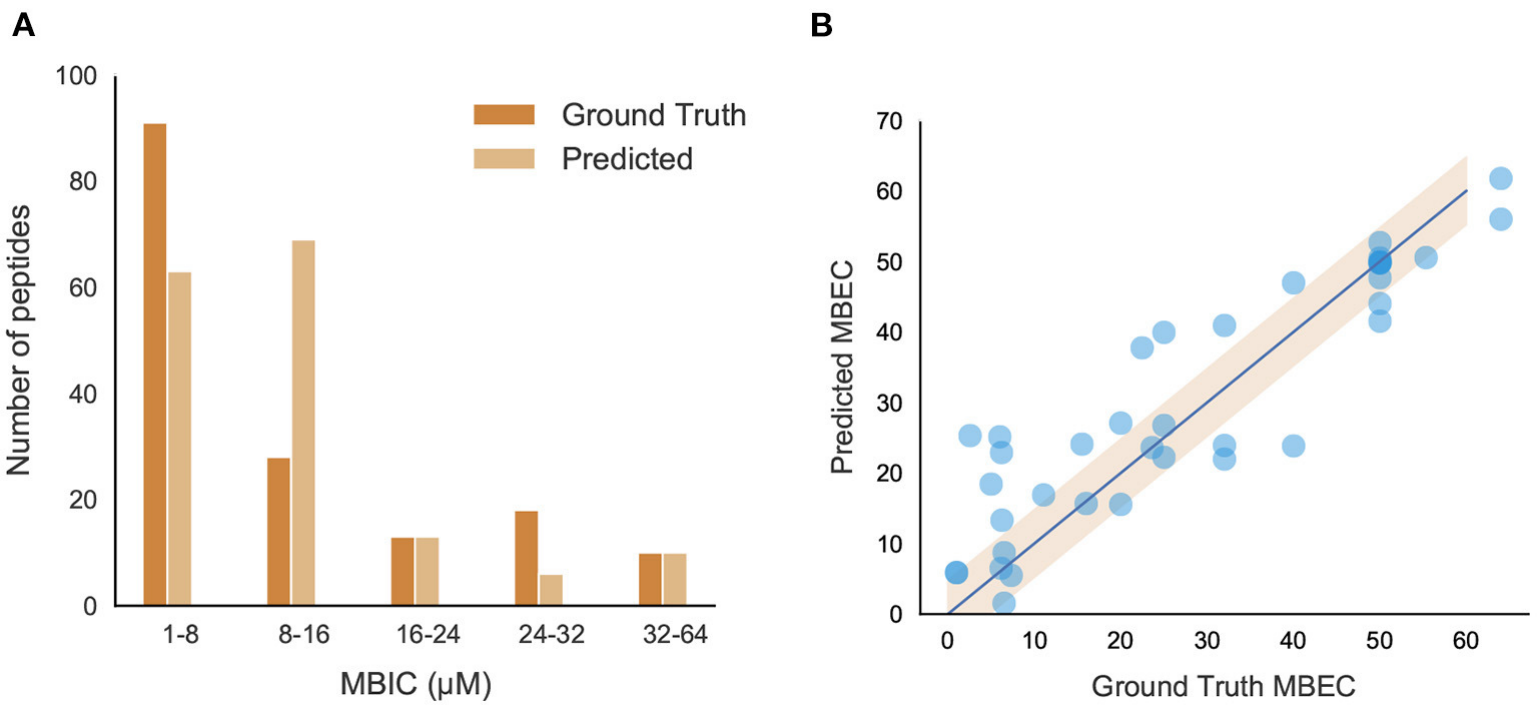
Performance and characteristics of peptides with MBIC/MBEC values. **(A)** Comparison between original and predicted MBIC values using the best regression model; **(B)** Performance of the best MBEC regression model. Best fit line (R^2^ = 0.832) and 95% confidence interval are shown.

The positive peptide dataset for the classification model contains 242 anti-biofilm peptides, 178 of which we were able to obtain MBIC values for. Although MBIC spanned from 0 to 640 μM, the data was largely skewed with approximately 80% of the values less than 64 μM and 52% of the values less than 20 μM. Given this imbalance, we trained an SVM to classify peptides above or below 64 μM, and a separate Support Vector Regression (SVR) model to predict the MBIC value of a peptide. Both models used an RBF kernel and a dataset consisting of only those peptides with MBIC values less than or equal to 64 μM. Each peptide consisted of 571 features and, due to this large dimensionality, feature selection was implemented using the *forward selection* algorithm to choose the most effective features. While iterating through forward selection, Root Mean Square Error (RMSE) for the training set peptides was minimized in the case of SVR and MCC was maximized in the case of SVM. Forward selection was halted upon 5 consecutive iterations where RMSE had not decreased or MCC increased. During feature selection, the samples were transformed into a lower dimensional space via Principal Component Analysis (PCA). Several hyperparameters were tuned, namely the regularization parameter (*C*) and kernel coefficient (γ) for the SVM/SVR models, and the number of principal components for the dimensionality reduction. We employed 5-fold stratified cross validation for classification and 5-fold cross validation for regression to ensure we trained a generic enough model that would not overfit the training set. For both models, a grid search between 0.001 to 1000 was used for both *C* and γ and the number of principal components spanned from 1 to the number of forward selection features. The best SVM model we found contained 9 features and was trained with parameters *C* = 10, γ = 950, and 6 principal components. The best SVR model contained 9 features as well and used parameters *C* = 45, γ = 40, and 8 principal components. The final model was able to achieve an MCC of 0.81 and an RMSE of 8.51 on the out-of-sample test sets. Figure 6A shows several ranges of MBIC values and the number of actual predicted MBIC values in each range.

##### 3.2.3.2. Models for the Prediction of Minimum Biofilm Eradication Concentration

We further evaluated the peptides and found only 57 from literature where the MBEC values have been reported. The analysis of peptides showed almost 85% of peptides having a sequence length <30. These peptides showed a high percentage of positively charged amino acids like arginine and lysine as well as aromatic amino acids like tryptophan. Additionally, secondary structure analysis showed a high percentage of helices present in those peptides (figures in Supplementary Note 4). After eliminating peptides with MBEC values greater than 64 μM, our dataset consisted of 42 peptides for further regression analysis.

A Support Vector Regression (SVR) model built with an RBF kernel was found to be the most effective model to predict MBEC values given the limited training dataset. The same dimensionality reduction methodology was used as in the MBIC models in addition to the same hyperparameter grid search. The best SVR model contained 12 features and used hyperparameters *C* = 900, γ = 20, and 7 principal components. It was trained using 5-fold cross-validation and the best performing model had an RMSE of 8.41. When ground truth MBEC values were plotted against the regression predictions, we found the R-squared value to be 0.832 (Figure 6B).

### 3.3. Classification of Novel Antibiofilm Peptides

We used our best machine learning models to predict peptides with potential for antibiofilm activity from diverse sources. We queried various peptide databases to identify peptides with antibiofilm activity. We searched 4,700 antimicrobial peptides, 74 anticancer peptides, and 212 antiviral peptides in the DRAMP database. We also considered more than 131,298 unique peptides from UniProt which have a sequence length of 4–80. After removing duplicates, we ran our classification model on 135,015 unique peptides from these varied sources, and selected 5468 unique peptides which were predicted as positive or hits by our model. The overall hit rate for this initial set of predicted peptides is 4.04% while the hit rate only from DRAMP database is 30.49%. This higher value is due to the inclusion of peptides with established antimicrobial activity in the antimicrobial databases, which is likely to be skewed for high antibiofilm activity.

**Figure 7:**
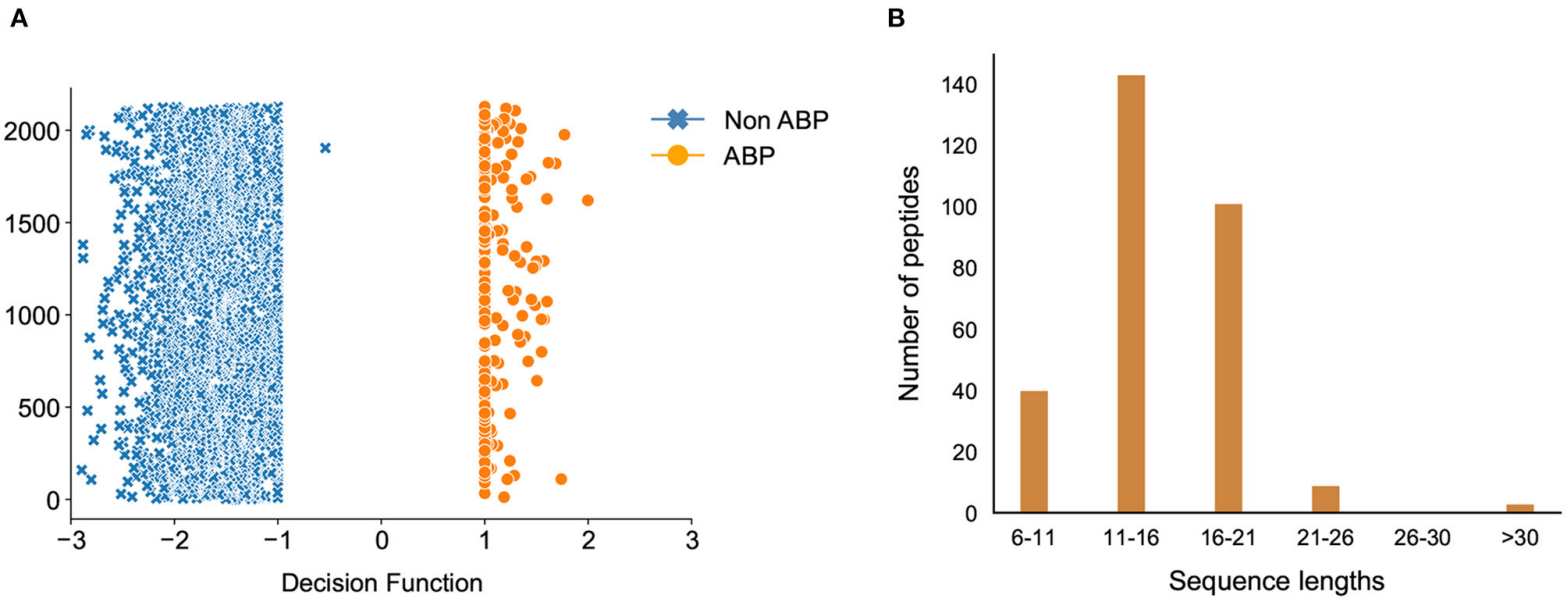
Classification of antibiofilm peptides. **(A)** Decision Function of the training data, decision function values >0.99 are the antibiofilm peptides whereas values <0 are the non-antibiofilm peptides; one antibiofilm peptide was misclassified by the model; **(B)** Distribution of predicted antibiofilm peptides based on their sequence lengths.

Due to the relatively large number of peptides selected by our classification model, we used the decision function of each of the peptides to narrow down the hits. The decision function estimates the sample position with respect to the discriminating hyperplane of the model. While training our model, we noticed that the peptides with decision function values higher than 0.99 were ABPs, whereas those with a negative decision function were non-ABPs (Figure 7A). More importantly, we noticed a clear discontinuity in the decision function as we transition from the positive to the negative dataset, which further showcases the effectiveness of our computational prediction model. Therefore, we used this confidence value as a filtering criteria to narrow down our list of peptides in the candidate set which were initially predicted as antibiofilm by our classification model and set a cut-off threshold of 0.99. As a result, we chose candidate peptides with decision function values higher than 0.99 as more likely to have antibiofilm activity, which further narrowed down the hits to 296 peptides or a hit rate less than 0.2% overall. A vast majority of these peptides have lengths between 6 and 21 amino acids (Figure 7B).

### 3.4. Prediction of Activity in Novel Antibiofilm Peptides

**Figure 8:**
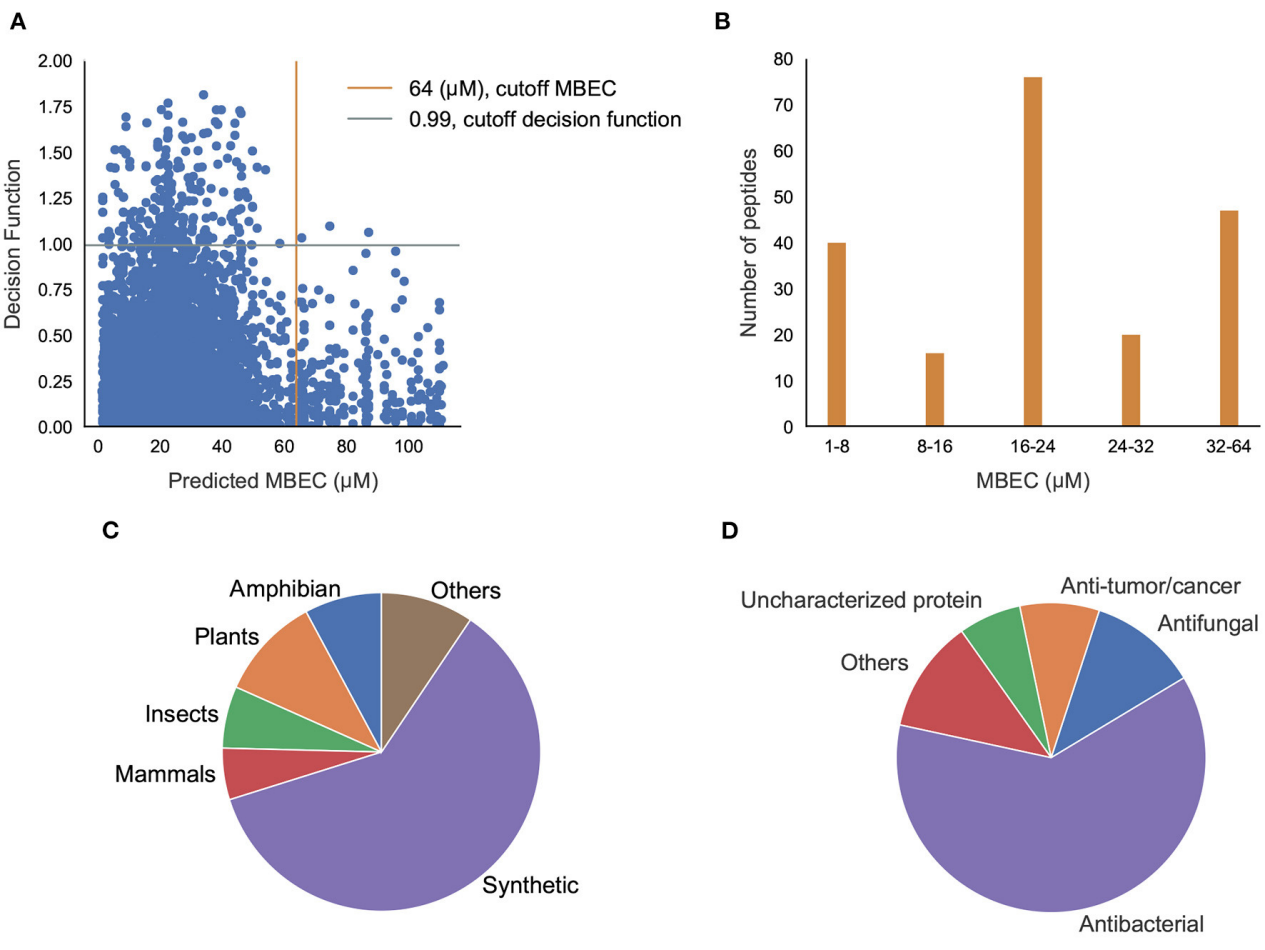
Prediction of antibiofilm activity using an integrated classification-regression scheme. **(A)** Decision Function of the classifier and Predicted MBEC from the regression model of the peptides; **(B)** Distribution of MBEC values in the 1-64 μM range; **(C,D)** Distribution of the peptides with predicted antibiofilm activity based the source **(C)**, or the function **(D)**.

Having classified potential ABPs, we used the regression models to predict the MBIC and MBEC values of the 296 peptides. Since we are interested in peptides that are efficacious against preformed biofilms, we used an operational cut-off of decision function ≥ 0.99, and MBEC ≤ 64 μM. We obtained 185 peptides (Supplementary Tables S6–S12 in Supplementary Note 5) of interest (Figure 8A). Among these peptides, 40 showed high effectiveness (MBEC between 1.0–8.0 μM) and another 48 peptides had comparatively lower effectiveness (MBEC more than 32 μM) (Figure 8B). We noticed that most of the peptides (67 peptides) had moderate predicted effectiveness between 16.0 and 24.0 μM. When we evaluated the source of the peptides, most of them were synthetic (116), and the naturally occurring peptides were from expected sources such as plants, amphibians, insects, and mammals (Figure 8C). We grouped the hits into those with known antimicrobial activity as archived in the DRAMP database (Supplementary Tables S6–S10 in Supplementary Note 5), and those that are not archived in the DRAMP database that were obtained from the UniProt database (Supplementary Tables S11–S13 in Supplementary Note 5). Of the 185 hits, 131 peptides were from the DRAMP database, and 54 were from the UniProt database. We also grouped the peptide hits based on their function. As expected, most of the peptides have some previously reported antibacterial properties. The hits also contained 19 peptides with anti-tumor/cancer activity, 26 peptides with antifungal properties, and 1 peptide with antiplasmoidal activity, suggesting potentially useful dual function therapeutics (Figure 8D).

Some of these peptide hits have already shown promise as antimicrobial agents (but not as antibiofilm agents) (Sahoo et al., 2021). The two anticancer peptides DRAMP03575 and DRAMP03829 are reported to exhibit an MIC of 16 μM and 10 μM, respectively, against *Escherichia coli* (Kang et al., 2019). The sperm protamine peptides from catshark and bat showed activity against 12 pathogens at a concentration of 0.01–20 mg/mL (Kim et al., 2015). Mastoparan-1 has an MIC value between 2 and 32 mg/L against *Staphylococcus aureus*, and is known to have antibacterial and antibiofilm properties (Memariani et al., 2018). Ponericins, peptides from the Ponerine ant, have structural similarity with well-known antimicrobial and antibiofilm peptide cecropins (Orivel et al., 2001). While no known antimicrobial activity has been listed for the histamine releasing peptide from the oriental hornet, studies are ongoing for alpha-conotoxin obtained from cone snails, mainly used in pain management, to establish its antimicrobial activity (Ebou et al., 2021). An analog, ω-conotoxin MVIIA shows the peptide is effective against *Candida kefyr* and *Candida tropicalis* with moderate MIC values between 28 and 40 μM but it was not effective against any bacterial assay up to 500 μM (Hemu and Tam, 2017).

### 3.5. Prediction of Secondary Structure of Novel Antibiofilm Peptide Hits

We sought to understand the possible mechanisms of action of newly found potential antibiofilm hits in the top quadrant of (Figure 8A). Since we were interested in peptides with previously unreported antimicrobial activities, and therefore novel, we focused on the 54 peptides from the UniProt database. Even amongst these 54 peptides, 14 peptides have been reported to have antimicrobial activities as per the UniProt annotation although they were not listed in any of the antimicrobial databases. For instance, the peptides P0CF03, P82420, and C0HK43 have been reported to have antimicrobial properties as well non-hemolytic properties, and anticancer properties (C0HK43) suggesting that these peptides may serve as good antibiofilm candidates. Other peptides such as P30259, P0C424, C0HLM2, Q9U8M9, A0A1C8YA26, P85874, and Q16228 are not reported to have any antimicrobial/antibacterial activity as per their UniProt annotation. Of the 40 peptides that do not have any annotations or references to antimicrobial activity, we found 5 peptides from the mastoparan group, 3 peptides from the poneritoxin group, 2 peptides each from the conotoxin, lasioglossin and protamine groups, and 17 uncharacterized peptides that are mostly derived from plant sources. Of note, our positive dataset did not contain any peptides from the mastoparan, poneritoxin, conotoxin, lasioglossin or protamine groups indicating that these are indeed novel hits. Since the function of peptides from the mastoparan, ponericin, and conotoxin groups is to protect the host organism, they belong the category of host defense peptides (HDPs). Interestingly, we also obtained non-host defense peptide hits which have varied functions—DNA intercalation (protamine), intemediate filaments in neurons (peripherin), and metabolic enzymes (alcohol dehydrogenase, beta-amylase).

**Figure 9:**
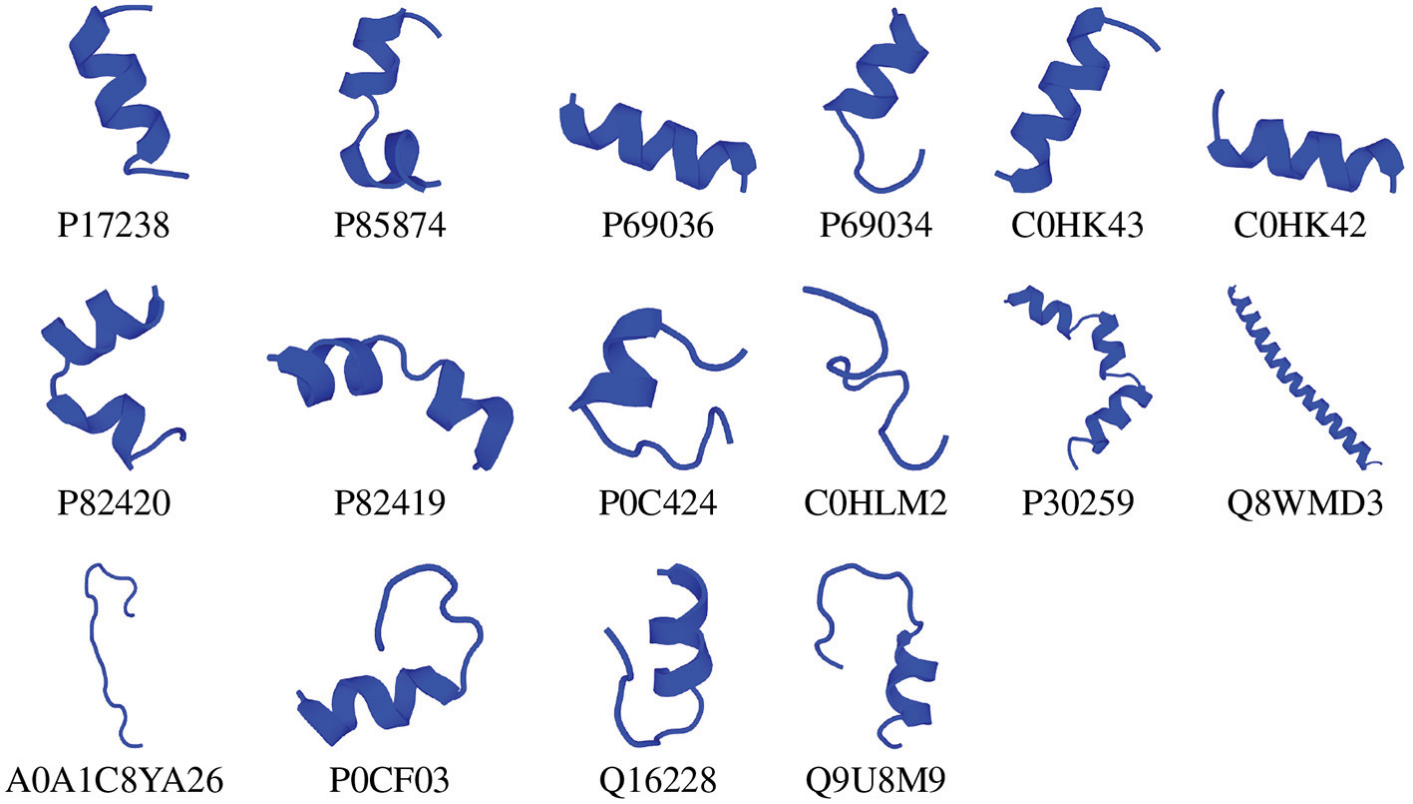
3D structures of previously characterized potential antibiofilm peptides. The 3D structures were predicted using the PEP-FOLD 3 server. All structures are depicted with N-terminal at the top, and C-terminal at the bottom.

**Figure 10:**
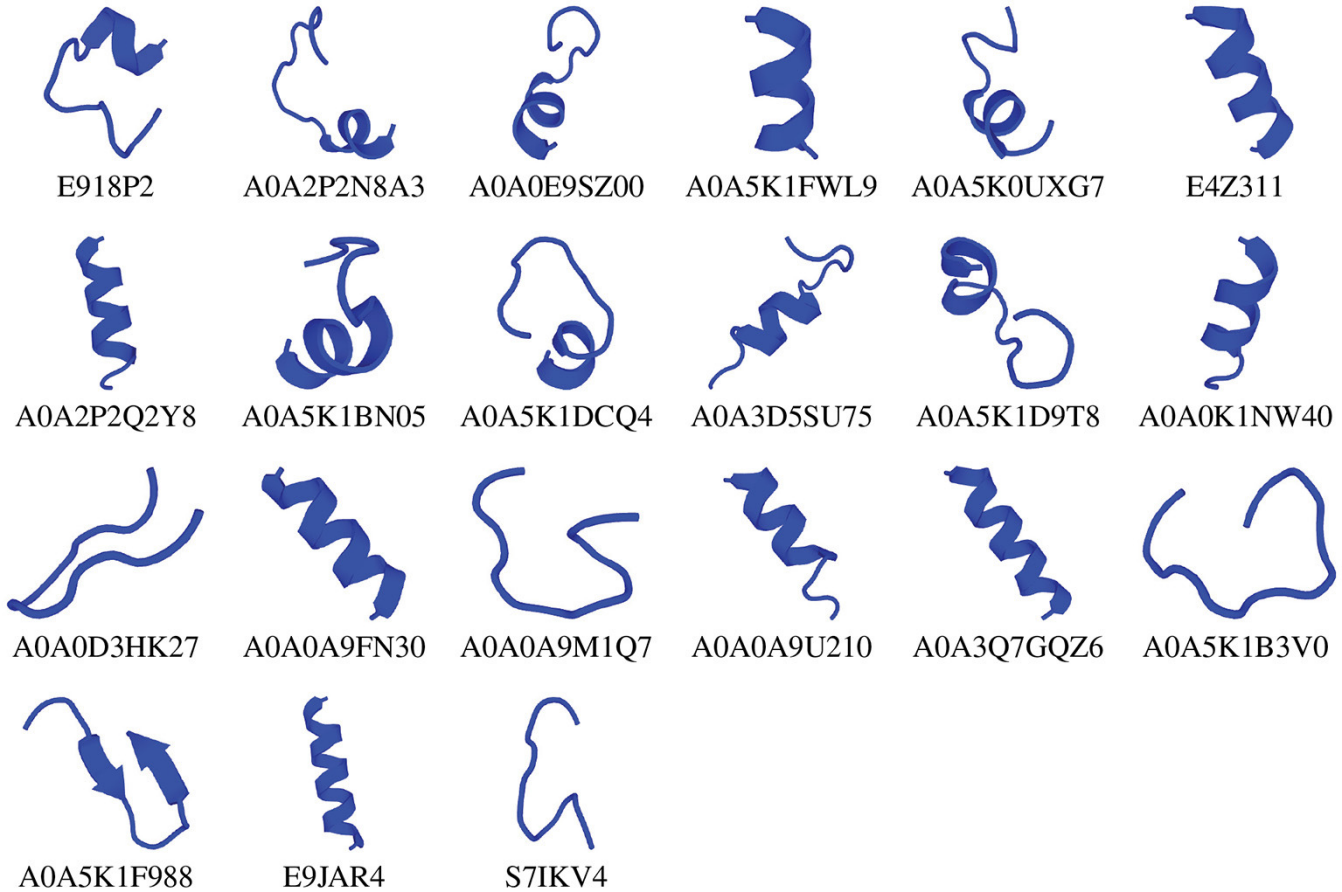
3D structures of previously uncharacterized potential antibiofilm peptides. The 3D structures were predicted using the PEP-FOLD 3 server. All structures are depicted with N-terminal at the top, and C-terminal at the bottom.

We chose the 37 novel peptides for further analysis, and their decision function and predicted MBEC values are listed in Table 1. In this Table, the first 16 peptides have been characterized previously, and the next 21 peptides have not been characterized (i.e., listed as uncharacterized in the Uniprot database). Next, we predicted the secondary structures of these peptides using the PEP2D server (Singh et al., 2019) and PEP-FOLD3 server (Lamiable et al., 2016). Figures 9, 10 show the predicted 3D structures, and Supplementary Figures S3, S4 in Supplementary Note 5 show the predicted 2D structures of previously characterized and uncharacterized peptides, respectively. Most peptides show helical or coil structures. Specifically, the mastoparan, poneritoxin, and lasioglossin peptides show helical structure, while the conotoxin peptides have a purely coil structure (Supplementary Figure S3 in Supplementary Note 5). The peptides not belonging to any specific class or uncharacterized peptides as per Uniprot annotation also showed mostly helical or coil structures with only one or two peptides folding as sheets (Supplementary Figure S4 in Supplementary Note 5). Using the DisEMBL predictor from JalView, we also observed that three hits (A0A0D3HK27, A0A2P2N8A3, E9I8P2) are likely to be highly mobile and not fold to a stable structure.

**Table 1:** Antibiofilm peptide hits.

**Name**	**Type**	**Sequence**	**Decision function**	**Predicted MBEC (μM)**
P17238	Mastoparan	INLKAIAALVKKVL	1.142	22.669
P85874	Mastoparan-like-peptide PMM2	INWKKIASIGKEVLKAL	1.109	22.679
P69036	Mastoparan	INWLKLGKAVIDAL	1.065	22.679
P69034	Mastoparan	INWLKLGKKVSAIL	1.011	22.679
P82420	U1-poneritoxin-Ng3g	GLVDVLGKVGGLIKKLLPG	1.326	27.710
P82419	M-poneritoxin-Ng3f	GLVDVLGKVGGLIKKLLP	1.299	28.502
P0CF03	U1-poneritoxin-Da2a	FLGGLIGPLMSLIPGLLK	1.001	19.722
C0HLM2	Alpha-conotoxin	SGCCKHPACGKNRC	1.729	39.130
P0C424	Conotoxin	CCAPSACRLGCRPCCR	1.348	2.967
C0HK43	Lasioglossin	VNWKKILGKIIKVAK	1.252	3.071
C0HK42	Lasioglossin	VNWKKVLGKIIKVAK	1.169	3.071
P30259	Protamine	GCKKRKARKRPKCKKARKRP-KCKRRKVAKKKC	1.278	4.289
Q8WMD3	Protamine	MARYRRCRSRSRCRRRRRRCH-RRRRRCCRRRRRRRACCRRYRCRRR	1.043	21.567
Q16228	Peripherin	WRWRRACRRPGRPFWRV	1.533	61.163
Q9U8M9	Alcohol dehydrogenase	AGLGGIGLDTNREIVKSGPK	1.079	20.531
A0A1C8YA26	beta-amylase	FLGCRVQLAIKISGI	1.072	22.358
A0A0A9FN30	Uncharacterized	MFRSLRKELKSKLL	1.052	22.845
A0A0A9M1Q7	Uncharacterized	MGRKFKWKLWT	1.448	14.309
A0A0A9U210	Uncharacterized	MTRIRRRRRHLLLLR	1.066	22.612
A0A0D3HK27	Uncharacterized	MFGGSGPLKLL	1.027	22.008
A0A0E9SZ00	Uncharacterized	MCTRWRVLLTCVRRR	1.299	28.711
A0A0K1NW40	Uncharacterized	KAIALALGKSGCK	1.226	22.678
A0A2P2N8A3	Uncharacterized	MGGKSDFRFCHVKKKVL	1.082	11.138
A0A2P2Q2Y8	Uncharacterized	MLKLWLRIKLLRKAL	1.225	35.601
A0A3D5SU75	Uncharacterized	PCPCGSGKKYKHCHGKLS	1.040	1.854
A0A3Q7GQZ6	Uncharacterized	GLAYRLVNLHFCKTKR	1.073	22.656
A0A5K0UXG7	Uncharacterized	ALLKSKPKLLRSGL	1.662	22.694
A0A5K1B3V0	Uncharacterized	VIRIGCKWKRTA	1.416	6.260
A0A5K1BN05	Uncharacterized	LGCGHGLPGIFACLK	1.030	3.071
A0A5K1D9T8	Uncharacterized	AKALGKRLRIKGRFQS	0.999	61.036
A0A5K1DCQ4	Uncharacterized	LGCGHGLPGIYACLK	1.233	3.071
A0A5K1F988	Uncharacterized	EKFKIHKSGKRWM	1.062	46.811
A0A5K1FWL9	Uncharacterized	FRARLLRTAFR	1.115	22.722
E4Z311	Uncharacterized	IKGILLRKIIKVR	1.138	35.514
E9I8P2	Uncharacterized	MLKKFLGKSGRRILR	1.173	8.156
E9JAR4	Uncharacterized	KLVLRRILALCIIAVCK	1.259	22.923
S7IKV4	Uncharacterized	SGLFCKGCSKL	1.030	60.153

### 3.6. Alignment of Sequences to Identify Consensus Patterns

**Figure 11:**
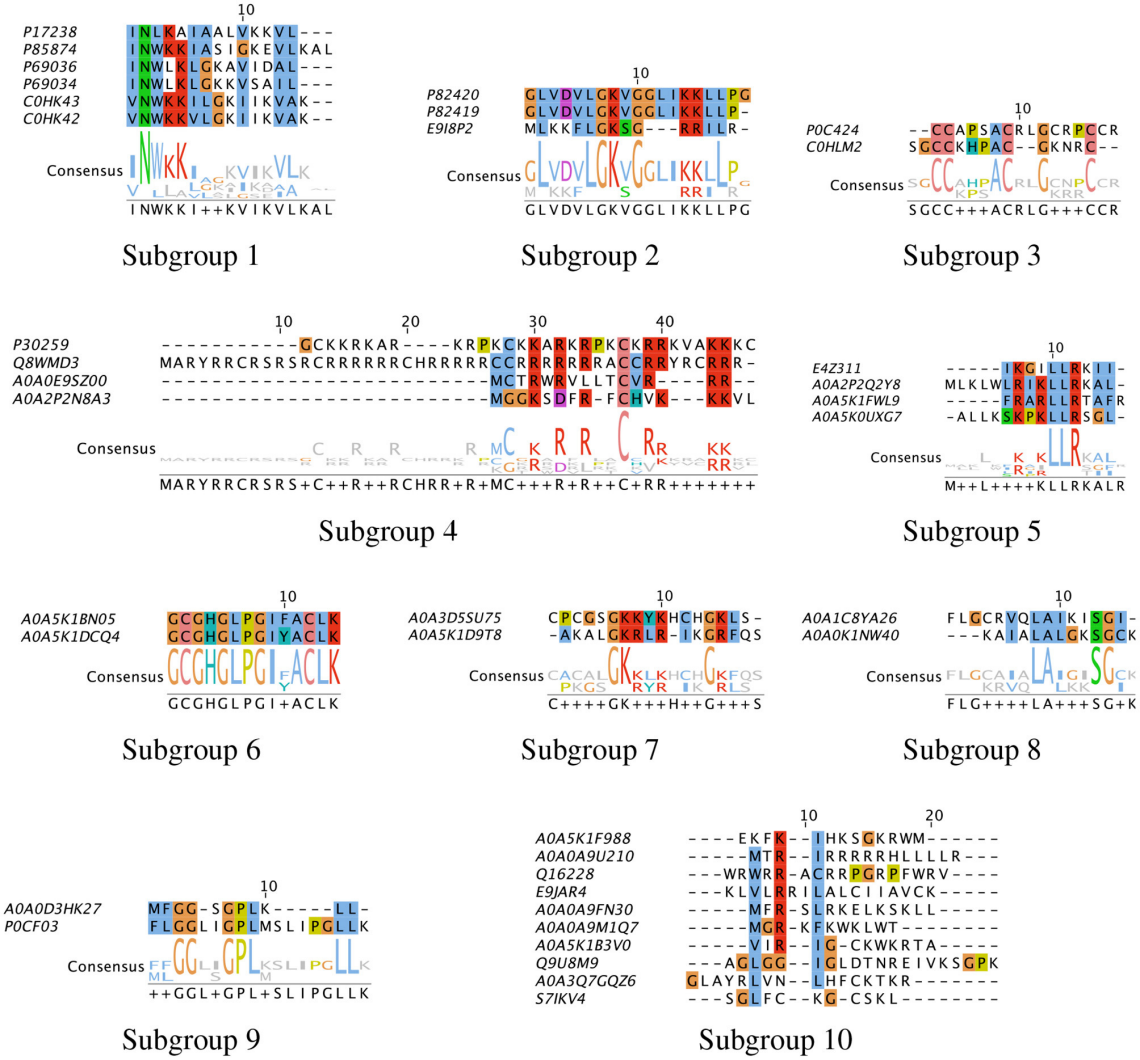
Multiple sequence alignment of peptide hits. The peptide hits listed in Table 1 were placed in Subgroups based on their BLOSUM62 neighbor in the phylogenetic tree, and Multiple Sequence Alignment with ClustalW was performed. The most conserved sequences for the Subgroups are: (1) INWKKI—V–VL; (2) L—LGK-G—KRLL; (3) CC—AC–G—C; (4) C-R-R-R–C-RR—RR; (5) LR-KLLR–L; (6) GCGHGLPGIFACLK; (7) GKRLR—GKL; (8) I-LAL–SG; (9) GG—GPL—LL. The default color scheme used as per ClustalX. Colorcode—blue: residue A, I, L, M, F, W, V, C; red: residue K, R; green: residue N, Q, S, T; magenta: E, D; yellow: residue P.

To characterize the unique sequences or common functional motifs among the 37 peptide hits, we performed multiple sequence alignment and generated the phylogenetic relationship between the hits. Based on these results, the peptide hits were classified into subgroups to obtain consensus sequences. The results are shown in Figure 11. We found that previously characterized peptides grouped into four subgroups: Subgroup-1, –2, –3 and −4, consisting of mastoparan and lassioglossin, poneritoxin, conotoxin, and protamine peptides, respectively. The previously uncharacterized peptides were grouped in Subgroups-5 through –9. The remaining hits that did show significant alignment with any other hit were placed in Subgroup-10. Some of the uncharacterized peptides aligned well with previously characterized peptides: E9I8P2 aligned with the poneritoxin group, and A0A0E9Z00 and A0A2P2N8A3 aligned with the protamine group. The common functional motifs were diverse but do not contain any anionic residues, and most commonly contain R, K, L, I, and G. We found sequences that were either R- or K-rich (Subgroup-1, −2, −4, −5, and −7), C-rich (Subgroup-3), I- or L-rich (Subgroup-1, −2, −5, −8 and −9). Interestingly, some of the common functional motifs, such as those from Subgroup-3, and −9, are unlike what is typically observed in AMPs.

Using helical wheel diagrams, we visualized helical peptides and their hydrophobic moment in Supplementary Figure S5 in Supplementary Note 5. The helical wheel diagrams show that the mastoparan and lassioglossin peptides (Subgroup-1) and A0A2P2Q2Y8 (Subgroup-5) form a nearly perfect amphipathic helix with hydrophilic and hydrophobic amino acids on either side of the helix. Other peptides such as A0A0A9210, A0A3Q7GQZ6, and E9JAR4 form helices but do not show amphipathic character as indicated by the lower value of hydrophobic moment.

To obtain insights into the mechanisms of action of these peptide hits, we sought to compare the sequences of these peptides with those in the positive dataset with known low MBEC values (<64 μM). Upon sifting through the positive dataset, we identified 5 peptides (LL-37, coprisin, melittin, RT2 and 1018) that have unique sequences and also have well established antibiofilm activity with known mechanisms of action (Supplementary Tables S19, S20 in Supplementary Note 6). We performed sequence alignment of the 37 peptide hits (16 characterized peptides and 21 uncharacterized peptides) with these 5 antibiofilm peptides using Clustal Omega, and the pairwise alignment score is shown in Table 2. As representative examples, the sequence alignments for the peptide pairs with the highest pairwise alignment scores are presented in Supplementary Figure S6 in Supplementary Note 5. The alignment scores are relative. The scores depend on both the length of the sequences and the number of identical or similar amino acids in those sequences. For instance, the self-alignment scores for the 37-amino acid long LL-37 is 1850, while the same score for alignment with the first five amino acids of LL-37 is 260.

**Table 2:** Pairwise alignment scores of known vs. previously characterized potential antibiofilm peptides along with predicted secondary structure.

**Peptide Name**	**Melittin**	**LL-37**	**Coprisin**	**RT2**	**1018**	**Secondary structure**
**Q16228**	90	160	40	**230**	150	helix-coil
**P69034**	**200**	140	90	60	70	helix-coil
**P82420**	**210**	120	0	40	40	helix-coil-helix
**P0CF03**	**190**	120	10	20	30	coil-helix
**P69036**	**180**	50	90	60	50	helix
**C0HK43**	**170**	**170**	70	50	90	helix
**C0HK42**	**170**	160	70	50	90	helix
**C0HLM2**	10	40	**170**	30	20	coil
**P0C424**	20	0	**160**	110	40	coil
**P85874**	140	**160**	30	50	90	helix-coil-helix
**P82419**	**150**	140	0	40	50	helix-coil-helix
**P17238**	**130**	120	50	0	80	helix
**A0A1C8YA26**	**130**	40	**130**	100	80	coil
**P30259**	**110**	50	80	0	20	helix-coil-helix
**Q8WMD3**	80	40	60	60	**90**	helix
**Q9U8M9**	**90**	70	50	60	0	coil-helix

*High-low scores are denoted in blue-white-red scale; Most match with known peptide is highlighted in bold; Secondary structure predicted using the PEP-FOLD 3 as shown in Figure 9*.

Of the previously characterized peptides, the hits which have a helical structure encompassing the mastoparan, poneritoxin, and lasioglossin groups showed significant sequence similarity with the melittin and LL-37 peptides. Conotoxin peptides with coiled structure share sequence similarity with coprisin. Interestingly, peripherin shared relatively high sequence similarity with LL-37, 1018 and RT2, demonstrating the highest pairwise alignment score with RT2 amongst all the peptides tested in this work. On the other hand, we notice that a few of the peptide hits (Q8MWD3 and P30259-protamines, Q9U8M9-alcohol dehydrogenase) showed low sequence similarity to the five known antibiofilm peptide sequences. These three peptides have an acceptable MBEC predicted to be less than 20 μM.

Of the uncharacterized peptides (Table 3), a majority of the peptides shared sequences similar to either melittin or LL-37. Among others, A0A0K1NW40 and E9JAR4 showed higher sequence similarity to coprisin, A0A0A9M1Q7 and A0A5K1F988 showed higher sequence similarity to RT2, and E4Z311 showed similarity to the 1018 peptide. A few peptides (A0A5K1FWL9, A0A5K1D9T8, S71KV4, A0A2P2N8A3) showed poor alignment with any of the five known antibiofilm peptides. Of these five peptides, A0A2P2N8A3 and A0A5K1FWL9 are predicted to have low MBEC values. A0A2P2N8A3 is rich in lysine and hydrophilic residues while A0A5K1FWL9 is rich in arginine and hydrophobic residues, indicating the diversity in the sequences of the hits.

**Table 3:** Pairwise alignment scores of known vs. previously uncharacterized potential antibiofilm peptides along with predicted secondary structure.

**Peptide name**	**Melittin**	**LL-37**	**Coprisin**	**RT2**	**1018**	**Secondary structure**
**E9I8P2**	30	**230**	40	0	60	coil
**A0A0A9M1Q7**	50	130	30	**220**	60	coil
**A0A0K1NW40**	30	50	**200**	70	50	helix
**A0A5K1F988**	30	**190**	40	180	50	sheet
**E4Z311**	90	110	50	10	**190**	helix
**A0A5K1DCQ4**	**190**	20	70	60	30	coil
**A0A5K1BN05**	**190**	40	50	20	30	coil-helix
**A0A0A9U210**	**180**	90	50	10	150	helix
**A0A2P2Q2Y8**	120	90	10	80	**180**	helix
**A0A0E9SZ00**	**170**	60	90	90	90	coil-helix
**A0A0D3HK27**	**140**	80	30	10	90	coil
**A0A5K0UXG7**	**140**	70	110	80	10	coil-helix
**A0A5K1B3V0**	50	**150**	50	50	140	coil
**E9JAR4**	100	80	**150**	0	80	helix
**A0A3Q7GQZ6**	50	**140**	70	0	30	helix
**A0A3D5SU75**	40	70	**130**	20	20	coil-helix
**A0A0A9FN30**	60	**120**	50	20	60	helix
**A0A5K1D9T8**	20	**110**	10	20	80	coil
**S7IKV4**	50	**110**	90	20	40	coil
**A0A2P2N8A3**	40	60	**110**	20	40	coil
**A0A5K1FWL9**	30	**50**	**50**	40	0	helix

*High-low scores are denoted in blue-white-red scale; Most match with known peptide is highlighted in bold; Secondary structure predicted using the PEP-FOLD 3 as shown in Figure 10*.

The results from secondary structure prediction and sequence alignment analysis provide insights into the possible mechanisms of action of the novel antibiofilm peptides. Although poorly understood, the antibiofilm peptides work through a variety of mechanisms including membrane disruption, inhibition of motility, disruption of essential proteins, and interruption of genetic elements (Raheem and Straus, 2019). The interaction with cell membrane is naturally favored by the amphipathic helices which is commonly found in most antimicrobial peptides, and in antibiofilm peptides (Zeng et al., 2021). Therefore, we expect that peptides from Subgroup-1 and Subgroup-5 including mastoparan and poneritoxin peptides may show membrane disrupting activity similar to the AMP. The Subgroup-4 peptides with high overall positive charge may interact favorably with negatively charged membrane. The coiled peptides (conotoxin, peripherin and protamine) may elicit antibiofilm action through mechanisms different from membrane interactions, possibly by interfering with vital cellular processes through specific binding interactions. For instance, the 1018 peptide is believed to interact with the signaling molecule ppGpp, and LL-37 by interfering with several pathways including quorum sensing (Overhage et al., 2008; Wieczorek et al., 2010). Lastly, the biofilm environment is generally acidic with pH 5–6, which may promote a change in secondary structure depending upon the pI of the amino acids (Xiong et al., 2017). Therefore, while this work provides some insights into novel peptide sequences and their mechanisms, detailed genetic and molecular biofilm inhibition assays are necessary to confirm the proposed mechanisms or delineate the mechanisms of action of novel peptides identified in this study.

### 3.7. Significance and Limitations of the Models

Unlike many other machine learning applications for antimicrobial peptides, we focused on the smaller subset of antibiofilm peptides instead of the much larger set of antimicrobial peptides because most recalcitrant infections are due to biofilms. Therefore, the drug development strategy should focus on efficacy against the biofilm mode of growth rather than the planktonic mode. Our approach differs from the previous models of antibiofilm peptide discovery in many significant ways. First, our negative dataset was curated as peptides which are likely to directly or indirectly promote biofilm formation rather than randomly generated sequences. Second, our model is built on the idea that the antibiofilm peptides are rarer to find in nature than biofilm-inhibiting peptides. To mimic that concept, we used ten times more peptides in the negative dataset than in the positive dataset. We considered stratified sampling and ten-fold cross-validation to eliminate the overfitting problem due to an imbalanced dataset. Third, we used motifs that are unique to the antibiofilm peptides not as privileged information but as a discovered entity while cross-validating the training dataset. This unbiased approach not only improved the performance of the model but also enabled the identification of truly discerning motifs, which changed the performance compared to the “without motif” model. Fourth, we developed SVR-based regression models for the prediction of the efficacy, i.e., the MBIC and MBEC values of novel peptides that were classified as ABPs by our classification model. Fifth, we used sequence alignment and secondary structure predictions to predict putative mechanisms of action of antibiofilm hits. Lastly, our work identified two broad classes of peptides: those peptides that have previously known bioactivity but not antibiofilm activity, i.e., those that may be considered for drug re-purposing; and those peptides without any previously reported bioactivity, i.e., those that may be considered as novel drug candidates.

One important limitation of using MBIC/MBEC values from the literature is that the peptides were not all tested against a single organism or using a single experimental technique but against a wide range of microorganisms (gram positive and gram negative bacteria, fungi), and using both dilution, plate-based or other techniques. This key limitation notwithstanding, the regression model performed very well, with an RMSE of 10–25% of the mean MBIC/MBEC values, for most peptides in the training set. We expect the effectiveness of our model to increase given more adequate MBIC/MBEC measurements on a wide range of ABPs. As an independent validation of our regression model, the predicted MBIC of these peptides matched well with experimentally determined antimicrobial activity (MIC). For instance, the peptide C0HK43 is active against gram-negative bacteria at concentrations of 1–14 μM as reported in the DRAMP database (MIC 1.4 μM against *E. coli*, MIC 14.1 μM against *P. aeruginosa*) while our model predicted an MBIC/MBEC value between 1 and 8 μM. The identification of a number of host defense peptides, which are considered to be a physiologically relevant response to biofilms, through a mechanism-agnostic sequence search further bolsters confidence in our approach (Hancock et al., 2021). Despite these limitations, our work has clearly demonstrated not only the feasibility of our sequence-based and mechanism-agnostic machine learning pipeline to predict efficacious antibiofilm peptides, but has also unearthed the vast diversity in sequences that have the potential to eradicate biofilms. Our platform may also be easily expanded to incorporate features such as the various post-translational modifications which may enhance the antibiofilm activity of the peptide backbone (Wang, 2012).

## 4. Conclusions

In this work, we have developed a machine learning pipeline for the classification of antibiofilm peptides followed by the determination of their efficacy. Using this pipeline, we identified a small set of novel antibiofilm peptides by mining diverse peptide libraries, and evaluated the efficacy of the hits. The peptide hits comprised of both novel peptides and peptides with other reported functions. Classical bioinformatics approaches of sequence alignment showed that some of the peptide hits may act through known mechanisms of antibiofilm activity while some others may follow less understood mechanisms to confer antibiofilm activity.

The two-tiered model enabled the classification and prediction of antibiofilm activity. Our SVM-based model with 572 features performed exceptionally well with an MCC of 0.91, which is significantly higher than current models. The higher performance is due to considering the physicochemical properties and motifs along with the compositions of peptides. Consistent with previously published studies, our model showed that the ABPs are characterized by the abundance of positively charged amino acids K and R, and higher hydrophobicity due to W and I. Our SVR-based model predicted the efficacy of ABPs with high confidence. To our knowledge, no previous studies have attempted to predict peptide efficacy using machine learning approaches. To this end, we built a regression model using the MBIC and MBEC values curated from the literature that have experimentally determined these values. In this work, we were careful to distinguish the biological significance of MBIC and MBEC values, as the former represents efficacy against biofilm formation, and the latter represents efficacy against pre-formed biofilms. An antibiofilm peptide with a lower MBIC but high MBEC may not effectively eradicate pre-formed biofilm. For instance, Dermaseptin-AC is useful in the inhibition of biofilm (MBIC 32 μM) formed by *Staphylococcus aureus* but is not effective in eradicating preformed biofilm (MBEC 256 μM) (Gong et al., 2020). Therefore, in this work, we developed models to predict both MBIC and MBEC for ABPs. *In vitro* biofilm inhibition and *in vivo* virulence assays beyond the scope of this work are warranted to confirm the validity and translational potential of the peptide hits.

## Data Availability Statement

The datasets presented in this study can be found in online repositories. The names of the repository/repositories and accession number(s) can be found in the article/Supplementary Material. The source code and instructions may be accessed at: https://github.com/davidanastasiu/antibiofilm.

## Author Contributions

BB, AR, and DA designed the research, analyzed the data, wrote the manuscript, and prepared the figures. BB collected data from databases and literature, performed classification experiments. TD developed and optimized the regression model. All authors approved the final version of the manuscript.

## Funding

This work was supported by an award from the NIH (R15AI138146).

## Conflict of Interest

The authors declare that the research was conducted in the absence of any commercial or financial relationships that could be construed as a potential conflict of interest.

## Publisher's Note

All claims expressed in this article are solely those of the authors and do not necessarily represent those of their affiliated organizations, or those of the publisher, the editors and the reviewers. Any product that may be evaluated in this article, or claim that may be made by its manufacturer, is not guaranteed or endorsed by the publisher.
