# Supplementary material for: Identification of Distinct Characteristics of Antibiofilm Peptides and Prospection of Diverse Sources for Efficacious Sequences
Source: Front Microbiol. 2022 Feb 4;12:783284. doi: 10.3389/fmicb.2021.783284 (PMC8856603; doi:10.3389/fmicb.2021.783284)
Supplement: Supplementary file 1 [file Presentation_1.pdf]

Supplementary Material

1 DATA AVAILABILITY

The datasets generated and analyzed in this study, along with codes, can be found in the Antibiofilm repository at
github.com/davidanastasiu/antibiofilm.

2 DATASET STATISTICS

Table S1 presents the number of peptides for training, validation and out-of-sample test sets for both the positive and negative datasets. The table also contains details of the dataset used for training and evaluating the regression models.

Table S1. Dataset Distribution of Our Machine Learning Models

**Dataset**	**Type**	**Training**	**Validation**	**Out-of-Sample Test**
Classification	Positive	175	19	48
	Negative	1741	194	485
MBIC Classification	$\leq 64\mu M$	128	32	N/A
	$> 64\mu M$	14	4	N/A
MBIC Regression	$\leq 64\mu M$	128	32	N/A
	$\leq 64\mu M$	33	9	N/A
Candidate	135015			

3 CHARACTERIZATION OF PEPTIDES

Figure S1 presents the ten dipeptides with the highest composition percentage from the negative dataset. Interestingly, most of the dipeptides in the top ten set contain leucine, a non-polar amino acid.

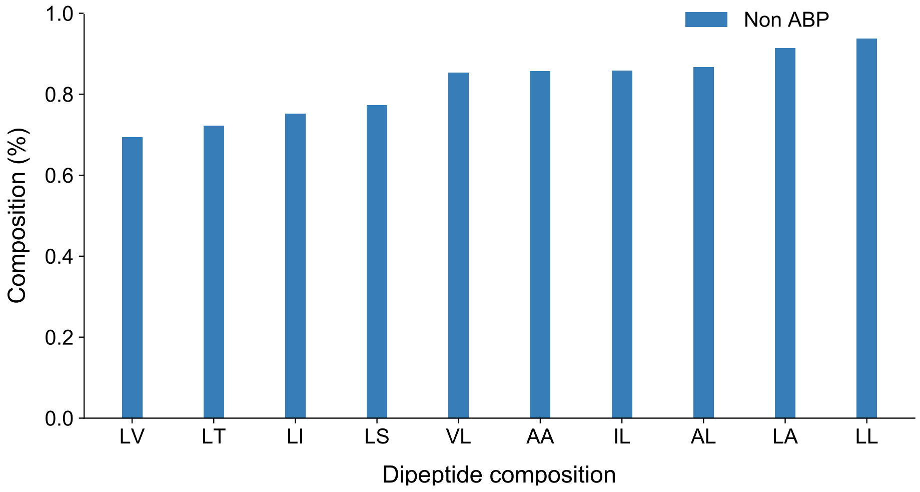

Figure S1. Dipeptide composition of the negative dataset; all the dipepetides contain the non-polar amino acid leucine;

4 PERFORMANCE OF MACHINE LEARNING MODELS

Table S2 presents results from our evaluation of different machine learning models based on individual features while Table S3 displays the performance of different models when we combine two features together. Finally, Table S4 showcases the performance of our models when we combine more than two features. Our best performing model combines the AAC, DPC, CTD and Motif features.

Table S2. Performance Evaluation of Different Machine Learning Techniques with Individual Features

Feature	Model	Sensitivity	Specificity	Accuracy	F1 Score	MCC
AAC	SVM	72.91	99.79	97.37	83.33	82.93
	RF	68.75	100	97.18	81.48	81.66
	XGBoost	75.11	99.38	97.18	82.75	81.76
DPC	SVM	85.41	98.35	97.17	84.35	82.99
	RF	72.91	99.17	96.81	80.45	79.24
	XGBoost	79.16	98.76	96.99	82.60	81.06
CTD	SVM	83.33	99.38	97.93	86.94	87.91
	RF	70.83	99.79	97.18	81.92	81.62
	XGBoost	85.41	98.96	97.74	87.23	86.02

Table S3. Performance Evaluation of Different Machine Learning Techniques with a Combination of Two Features

Features	Model	Sensitivity	Specificity	Accuracy	F1 Score	MCC
AAC & DPC	SVM	81.25	98.96	97.33	84.78	83.33
	RF	77.08	99.79	97.74	86.08	85.52
	XGBoost	81.25	99.38	97.74	86.66	85.76
DPC & CTD	SVM	77.08	99.79	97.74	86.04	85.52
	RF	70.83	100	97.37	82.92	82.97
	XGBoost	79.16	99.17	97.37	84.44	83.23
CTD & AAC	SVM	85.41	99.17	97.93	88.17	87.09
	RF	72.91	100	97.56	84.33	84.26
	XGBoost	79.16	99.58	97.74	86.36	85.56

Table S4. Performance Evaluation of Different Machine Learning Techniques with a Combination of Three or More Features

Features	Model	Sensitivity	Specificity	Accuracy	F1 Score	MCC
AAC & DPC & CTD	SVM (c=100, gamma=0.01)	85.41	98.96	97.94	88.42	87.29
	RF (n-estimator=100)	70.83	100	97.37	82.92	82.97
	XGBoost (n-estimator=100, gamma=0.5)	81.25	99.58	97.93	87.64	86.84
AAC & DPC & CTD & motif	SVM (c=150, gamma=0.05, Motif=ALL)	**85.48**	**99.79**	**98.49**	**91.11**	**90.53**
	RF (with Motif=BETTS-RUSSELL)	72.91	100	97.56	84.33	84.26
	XGBoost (with Motif=BETTS-RUSSELL)	85.41	99.38	98.12	89.13	88.20

Table S5. Performance Comparison of Our Method with the Dataset from Gupta et al. (2016)

Validation dataset performance	Specificity	Sensitivity	Accuracy	F1 Score	MCC
Reported in Gupta et al. (2016)	97.75	91.67	97.19	N/A	0.84
Achieved with our model	**99.71**	86.11	**98.46**	**91.17**	**0.90**

5 CHARACTERIZATION OF PEPTIDES FROM THE MBEC DATASET

We present the characteristics of the 57 peptides that were selected for training the regression model responsible for predicting the MBEC value of a candidate antibiofilm peptide.

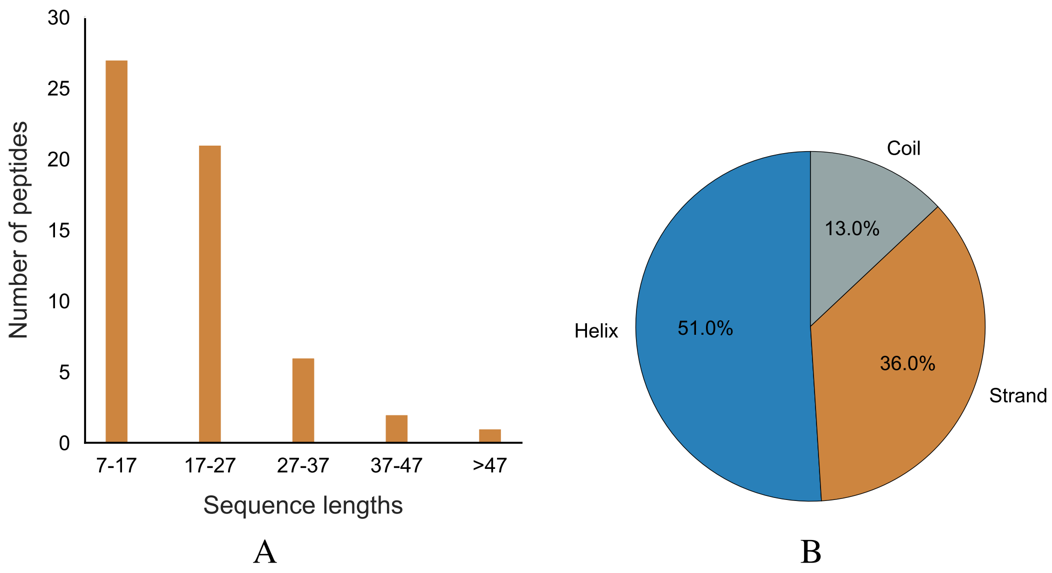

Figure S2. Performance and characteristics of peptides with MBIC/MBEC values; (A) Number of peptides with MBEC values in different sequence ranges; (B) Percentage of helices, strands and coils in secondary structure for peptides with MBEC values;

6 NEWLY FOUND ANTIBIOFILM PEPTIDES

6.1 Visualization

We have evaluated the 2D structures of the peptides using PEP2D server Singh et al. (2019).

We further evaluated the structure of the peptides with probable antibiofilm activity. We evaluated helical wheel structure (Figure S5) for the peptides which showed higher percentage of helices in secondary structure evaluation.

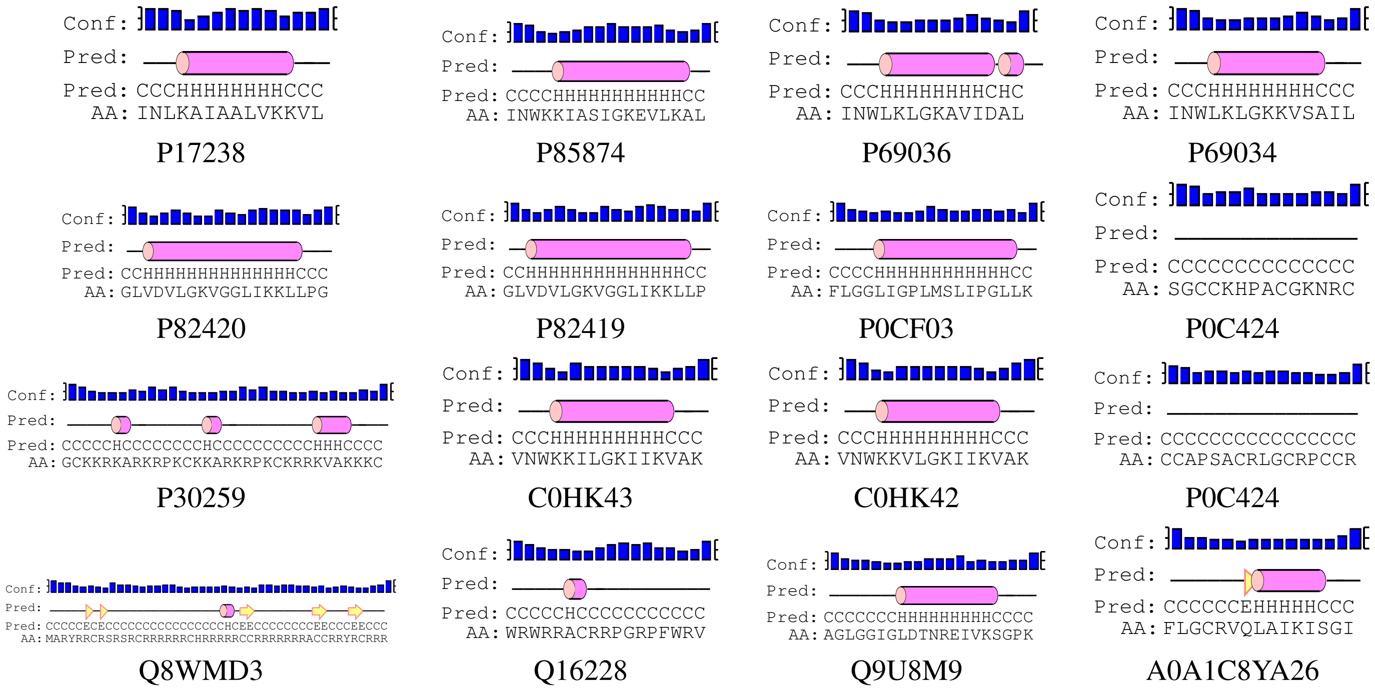

Figure S3. Predicted 2D structures of previously characterized peptides with potential antibiofilm activity.
The 2D structures were evaluated using the PEP2D server. The pink cylinders represent helix, yellow arrows represent sheet, and the black line is coil.

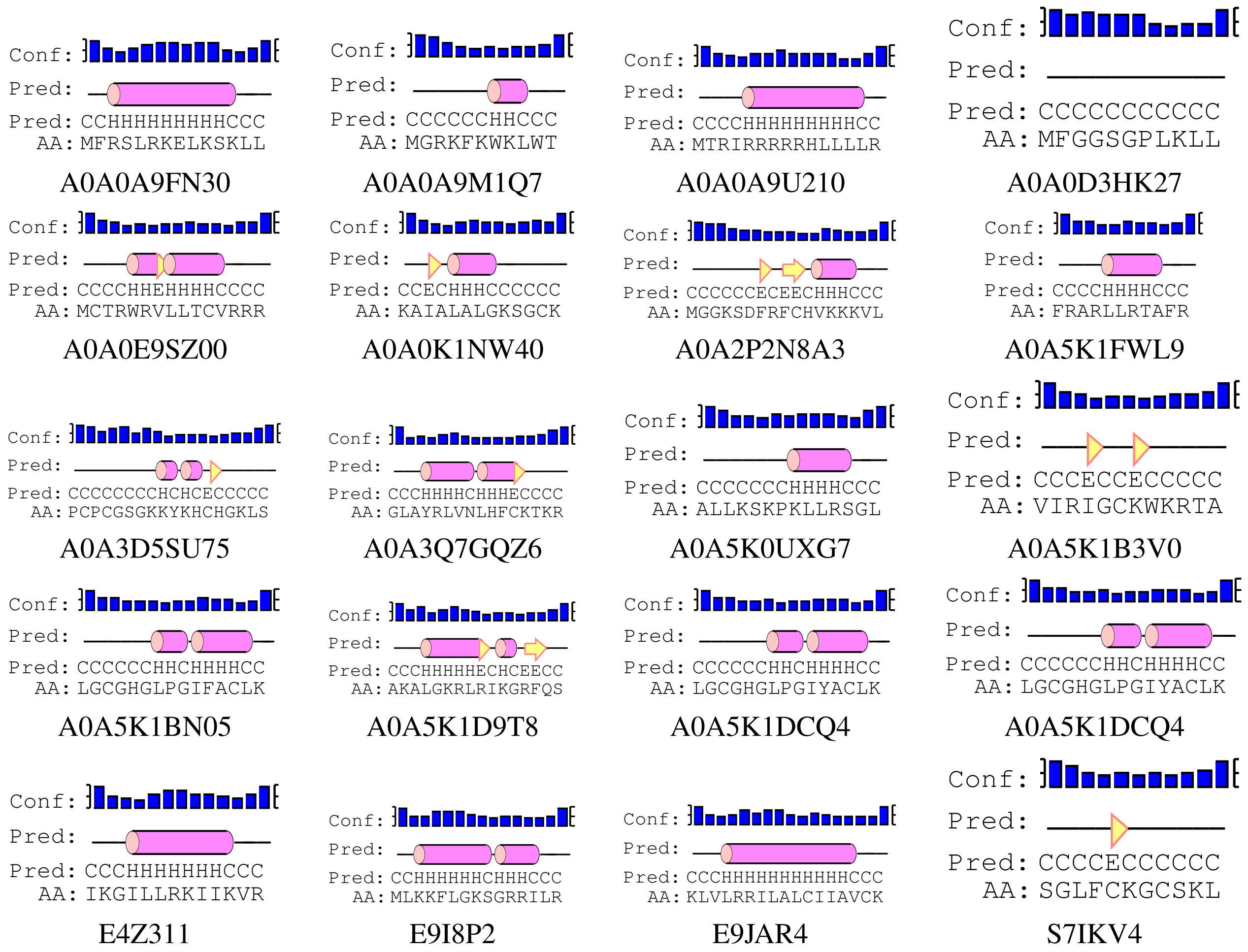

Figure S4. Predicted 2D structures of previously characterized peptides with potential antibiofilm activity.
The 2D structures were evaluated using the PEP2D server. The pink cylinders represent helix, yellow arrows represent sheet, and the black line is coil

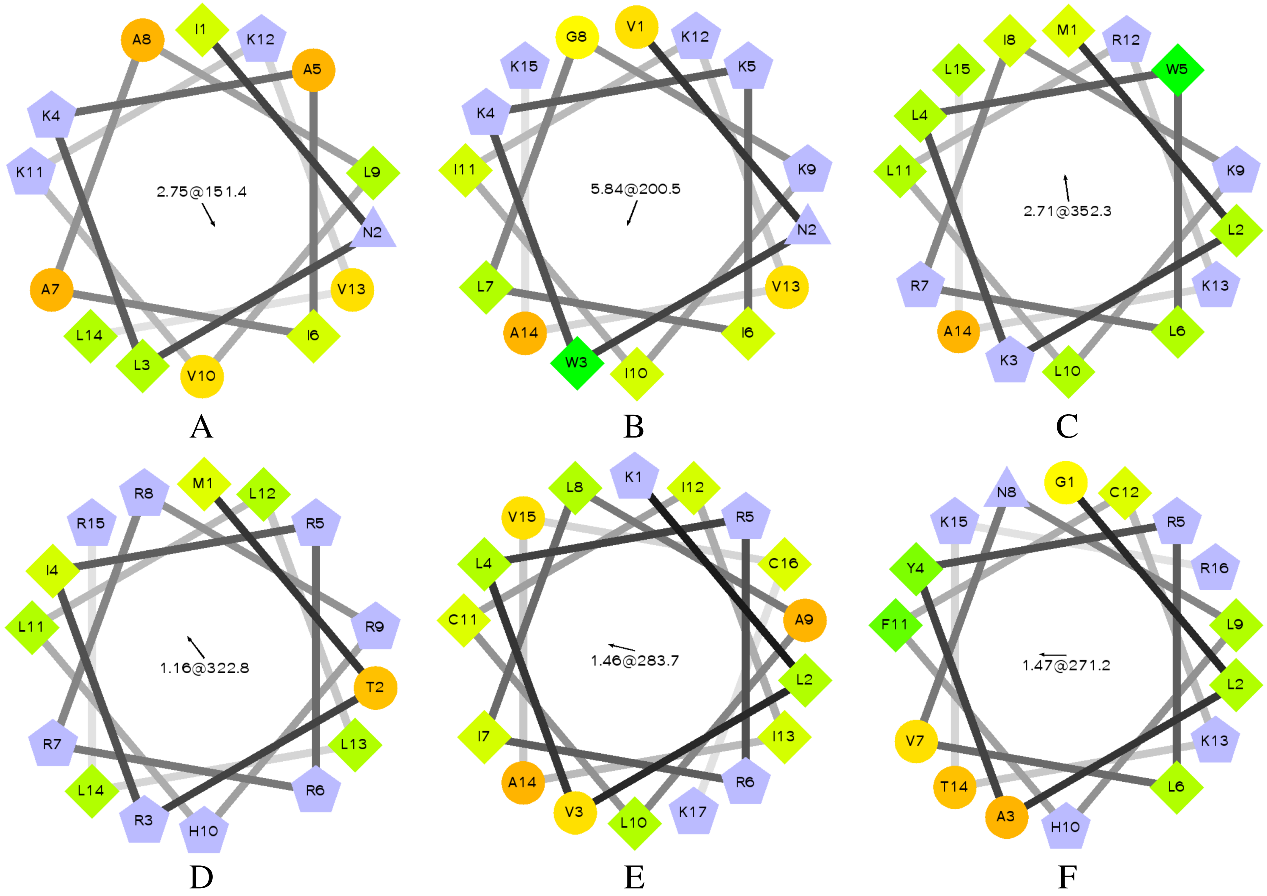

Figure S5. The helical wheel structures of a few newly found antibiofilm peptides; (A) P17238, Mastoparan; (B) C0HK43, Lasioglossin; Rest of the peptide marked as 'uncharacterized protein' (C) A0A2P2Q2Y8, (D) A0A0A9U210, (E) E9JAR4, (F) A0A3Q7GQZ6. Here, hydrophilic amino acids are shown in circles, hydrophobic as diamonds. Negatively charged amino acids are triangles, and positively charged are pentagons. The hydrophobic amino acids are green, and the green shade decreases to yellow as per decreasing hydrophobicity. Hydrophilic amino acids are in red and the amount of red decreases as per decreasing hydrophilicity. The highly charged amino acids are in light blue and non-polar amino acids are in dark red. The numbers indicate the hydrophobic moment and the direction of the moment. The wheel structures were obtained using the software created by Don Armstrong and Raphael Zidovetzki, version 1.4, 2009-10-20 Schiffer and Edmundson (1967); Armstrong and Zidovetzki (2009).

6.2 Alignment

We also analyzed a few newly found antibiofilm peptides against some well known antibiofilm peptides which already have an eradication effect on preformed biofilm. For example, we aligned human cathelicidin, LL-37, against the set of Mastoparan-like peptides from our list. The alignment was done using the Clustal default webservice Madeira et al. (2019). The alignment is displayed in Figure S6 using Jalview V2 Waterhouse et al. (2009).

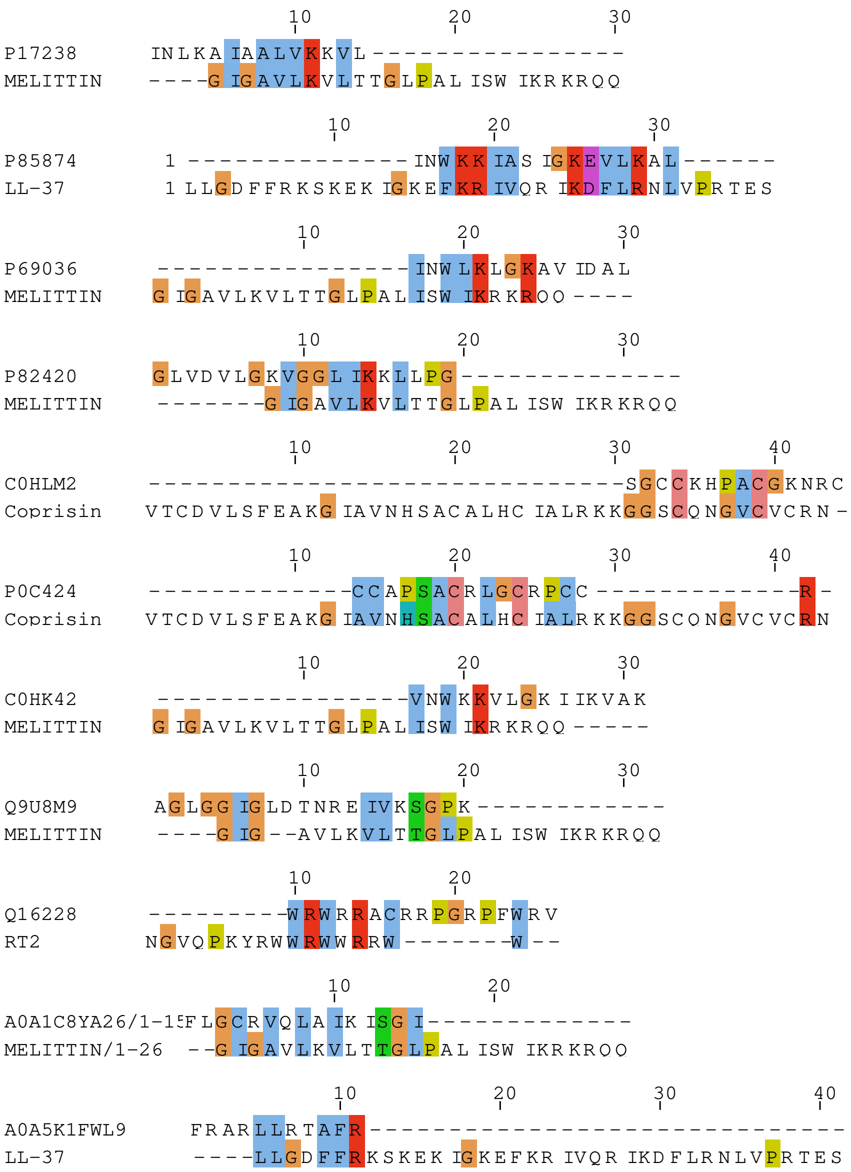

Figure S6. Pairwise sequence alignment of peptide hits with their closely matching known antibiofilm peptide. The default colour scheme used as per ClustalX
Colorcode – blue: residue A, I, L, M, F, W, V, C; red: residue K, R; green: residue N, Q, S, T; magenta: E, D; yellow: residue P.

6.3 Peptide List

The list of probable antibiofilm peptides from our pipeline are listed in Tables S6–S13. The tables contain peptide sequences and predicted MBEC values. We grouped the peptides in several MBIC value ranges.

Table S6. Newly Predicted Antibiofilm Peptides with MBIC Range 1–8 (μM) from the DRAMP database

**Name**	**Seq**	**Source**	**Predicted MBEC (μM)**
DRAMP04642	GIGKFLHSAGKFGKAFIGEIMKS	Synthetic	1.510
DRAMP02364	GFWGKLFKLGLHGIGLLHLHL	Mammals	1.617
DRAMP04663	AKRLKKLAKKIWKWL	Sheep	1.617
DRAMP01423	GLLSGILGAGKHIVCGLSGLR	Synthetic	3.071
DRAMP18605	VNWKKILAKIIKVVK	Synthetic	3.597
DRAMP18607	VNWKKILPKIIKVVK	Synthetic	4.332
DRAMP03983	KWKLFKKIPKFLHLA	Synthetic	4.332
DRAMP03862	RLRRIVVIRVRR	Frog	6.605
DRAMP01310	FLGLLPSIVSGAVSLVKKL	Synthetic	7.090
DRAMP04187	KWLKKLLKKLL	Synthetic	8.957
DRAMP03974	WKKIPKFLHLAKKF	Fish	9.853
DRAMP18601	VNWKKILAKIIKVAK	Synthetic	11.312
DRAMP18602	VNWKKILKKIIKVAK	Synthetic	11.854
DRAMP18603	VNWKKILPKIIKVAK	Synthetic	13.681
DRAMP03981	KWKLFKKIPKFLHLAK	Synthetic	20.981
DRAMP03850	FALALKALKKALKKLKKALKKAL	Scorpion	22.396
DRAMP04002	ILGKILKGIKKLF	Xenopus muelleri	22.471
DRAMP18560	IWRIFRRIFRIF	Synthetic	22.608
DRAMP03859	RLARIVVIRWAR	Synthetic	22.632
DRAMP03880	RRIIIRWRRI	Synthetic	22.668
DRAMP04318	IKWKKLLRAAKRIL	Synthetic	22.671
DRAMP18642	KWRWIW	Bacteria	22.674
DRAMP04297	LKALKKLAKKLKKLA	Synthetic	22.677
DRAMP04215	RRLFRRILRWL	Synthetic	22.677
DRAMP18514	KKLALHALKKWLHALKKLAHLALKK	Synthetic-De Novo	22.679
DRAMP04384	KIASIGKEVLKAL	Synthetic	22.679
DRAMP03702	LFGLIPSLIGGLVSAFK	Synthetic	22.679
DRAMP02910	LRRIIRKIIHIIKK	Synthetic	22.679
DRAMP02911	IRRIIRKIIHIIKK	Synthetic	22.725
DRAMP01621	IIGPVLGMVGSALGGLLKKI	Synthetic	22.837
DRAMP01626	IIGPVLGLVGSALGGLLKKI	Synthetic	24.947
DRAMP04125	SGKLWWRRKK	Frog	25.316
DRAMP03975	KWFKKIPKFLHLLKKF	*Bombina variegata*	25.316
DRAMP03973	WFKKIPKFLHLAKKF	Synthetic	25.568
DRAMP03979	KWKLFKKIPKFLHLAKK	Orange-legged leaf frog	25.616
DRAMP01807	FLPLVLGALSGILPKIL	Synthetic	29.105
DRAMP03977	WKKIPKFLHLLKKF	Synthetic	29.774
DRAMP03976	WFKKIPKFLHLLKKF	Synthetic	36.868
DRAMP18619	RKLRRLKRKIAHKVKKY	Synthetic	60.154

Table S7. Newly Predicted Antibiofilm Peptides with MBIC Range 8–16 (μM) from the DRAMP Database

**Name**	**Seq**	**Source**	**Predicted MBEC (μM)**
DRAMP02474	KFFRKLKKSVKKRAK	Spider	2.397
DRAMP18600	NVWKKILGKIIKVAK	Synthetic	2.462
DRAMP18568	VSWKKSLGKIIKVVK	Synthetic	3.071
DRAMP18596	NVWKKVLGKIIKVAK	Synthetic	3.071
DRAMP04228	GRFRRLGRKFKKLFKKYGP	Synthetic	3.071
DRAMP03972	KWFKKIPKFLHLAKKF	Scorpion	3.236
DRAMP02963	GRFRRLRKKTRKRLKKIGKV	Synthetic	4.011
DRAMP01126	ILGPVISKIGGVLGGLLKNL	Synthetic	4.332
DRAMP03570	LLGDFFRKSKEKIGKEFKRIVQR	Bovine	4.719
DRAMP03967	KWKLFKKIPKFLHLAKKF	Synthetic	4.953
DRAMP03969	KWKLFKKISKFLHLAKKF	Synthetic	4.953
DRAMP03970	WKLFKKIPKFLHLAKKF	Synthetic	6.610
DRAMP03971	FKLFKKIPKFLHLAKKF	Synthetic	10.699
DRAMP18563	KSLRRVWRSWR	Synthetic	10.791
DRAMP03826	KNLRRIIRKIIHIIKKYG	*Odorrana grahami* (Frog)	15.718
DRAMP04310	ELAKKALKALKKALKSAR	Synthetic	16.091
DRAMP04311	ELAKKALRALKKALKSAK	Pig	19.720
DRAMP03227	GLFGKLIKKFGRKAISYAVKKARGKH	Synthetic	19.996
DRAMP03980	KWKLFKKIPLHLAKKF	Toad	21.695
DRAMP03923	KWKLFKKIGIGAVLKVLT	Synthetic	22.608
DRAMP04260	LALLKVLLRKIKKAL	Synthetic	22.645
DRAMP18562	KSLVRRWRSRW	Synthetic	22.645
DRAMP18597	VNWKKVLAKIIKVAK	Synthetic	22.659
DRAMP18598	VNWKKVLKKIIKVAK	Synthetic	22.659
DRAMP18599	VNWKKVLPKIIKVAK	Synthetic	22.668
DRAMP03963	KWKLFKKIKFLHSAKKF	Synthetic	22.671
DRAMP03968	KWKLFKKILKFLHLAKKF	Synthetic	22.676
DRAMP03984	KWKLFKKIPLAKKF	Synthetic	22.679
DRAMP18570	VNRKKILGKSIKVVK	Synthetic	22.679
DRAMP18502	WKSYVRRWR	Synthetic	22.679
DRAMP04018	ALYKKFKKKLLKSLKRLG	Bacteria	22.679
DRAMP04003	ILGKIWKIKKLF	Synthetic	22.679
DRAMP04376	INWLKLGKKIISAL	Synthetic	22.705
DRAMP18507	FSGGNCRGFRRRCFCTK	Synthetic	22.725
DRAMP04614	AVNIPFKVHFRCKSIFC	Synthetic	22.837
DRAMP03865	RWWKIWVIRWWR	Synthetic	22.837
DRAMP18567	VNWKKILGKSIKVSK	Synthetic	23.595

Table S8. Newly Predicted Antibiofilm Peptides with MBIC Range 8–16 μM from the DRAMP Database (Cont.)

Name	Seq	Source	Predicted MBEC (μM)
DRAMP04664	AKRLKKLAKKIWKWK	Human	24.958
DRAMP04051	RICRIVVIRCIR	Human	25.040
DRAMP03978	KWKLFKKIPFLHLAKKF	Synthetic	25.219
DRAMP03925	KWKLFKKGAVLKVLT	Synthetic	25.595
DRAMP03768	ILSAIWSGIKS	Synthetic	25.595
DRAMP03858	RLARIVKIRVAR	Synthetic	25.595
DRAMP03982	KWKLFKKIPHLAKKF	Synthetic	28.344
DRAMP18564	KWLRRVWRWWR	Snake	35.077
DRAMP18565	KRLRRVWRRWR	Synthetic	39.599
DRAMP03964	KWKLFKKIPKFLHSAKKF	Synthetic	39.927
DRAMP03924	KWKLFKKGIGAVLKV	Synthetic	44.372
DRAMP03575	FKRIVQRIKDFLR	AnTomato	48.038
DRAMP02872	GRFKRFRKKFKKLFKKLS	Synthetic	50.828
DRAMP18501	WKSYVRRWRS	Synthetic	53.153
DRAMP03829	GLKKLLGKLLKKLGKLLLK	Synthetic	54.066
DRAMP18499	WKSYVRRWRSR	Synthetic	63.560

Table S9. Newly Predicted Antibiofilm Peptides with MBIC Range 16–32 (μM) from the DRAMP Database

Name	Seq	Source	Predicted MBEC (μM)
DRAMP03777	IWSAIWSGIKGLL	*Urodacus yaschenkoi* (scorpion)	35.514
DRAMP04390	INWKKGKEVLKAL	Synthetic	22.679
DRAMP03966	KLKLFKKIGIGKFLHSAKKF	Synthetic	7.220
DRAMP02914	RICRIIFLRVCR	Sheep	14.287
DRAMP04377	INWLKLGKKLLSAL	Synthetic	22.679
DRAMP04113	KWKSFIKKLASKFLHSAKKF	Synthetic	22.680
DRAMP04115	KWKSFIKKLTKKFLHSAKKF	Synthetic	45.279
DRAMP04127	RGKRWWRRKK	Synthetic	49.900
DRAMP04054	RLCRIVWVIRVCR	Synthetic	47.017
DRAMP18401	ILSAIWSGIKGLL	Scorpion	35.514
DRAMP03965	KAKLFKKIGIGKFLHSAKKF	Synthetic	5.068
DRAMP04102	KWKSFIKKLTSKFLHLAKKF	Synthetic	25.740
DRAMP04103	KWKSFIKKLTSKFLHSAKKF	Synthetic	45.279
DRAMP04104	KFKSFIKKLTSKFLHSAKKF	Synthetic	45.279
DRAMP04106	KWKSFKKKLTSKFLHSAKKF	Synthetic	45.279
DRAMP04119	KWKSFIKKLTSKFLHSKKKF	Synthetic	45.279
DRAMP04112	KWKSFIKKLLSKFLHSAKKF	Synthetic	22.380
DRAMP18723	KWKLFKKI	Moth	48.126
DRAMP18566	VNWKKSLGKSIKVVK	Synthetic	4.953
DRAMP01134	ILGPVIKTIGGVLGGLLKNL	Toad	19.866

Table S10. Newly Predicted Antibiofilm Peptides with MBIC Range >32 (μM) from the DRAMP Database

Name	Seq	Source	Predicted MBEC (μM)
DRAMP04343	IGKLFKRIVKRILKFLRKL	Synthetic	35.719
DRAMP04126	SGKRWWRRKK	Synthetic	49.900
DRAMP04339	IGKKFKRIVQRIKKFLRKL	Synthetic	3.556
DRAMP04340	IGKKFKRIVKRIKKFLRKL	Synthetic	3.556
DRAMP04345	IGKKWKRIVKRIKKFLRKL	Synthetic	3.556
DRAMP04346	IGKKFKRIVKRIKKWLRKL	Synthetic	3.556
DRAMP18614	VRRFAWWWAFLRR	Synthetic	22.940
DRAMP03876	RRWWWRWRRW	Synthetic	49.900
DRAMP03877	KKWWWKWKKW	Synthetic	49.900
DRAMP03878	RRWWRRWRRW	Synthetic	49.900
DRAMP03879	RRFFFRFRRF	Synthetic	49.900
DRAMP18457	KRWWKWWRRC	Synthetic	15.828
DRAMP18508	KFAKKFKWFAKAAFKFFKK	Synthetic	49.900
DRAMP18643	KWWWRW	Synthetic	49.900
DRAMP04001	ILGKIWKGIKSLF	Synthetic	7.590
DRAMP04069	CFKFKFKFGSGFKFKFKFC	Synthetic	22.791
DRAMP04070	CWKWKWKWGSGWKWKWKWC	Synthetic	22.791
DRAMP03869	RRWVIWRR	Synthetic	40.529
DRAMP18558	FIKRIARLLRKIF	Synthetic	34.455

Table S11. Newly Predicted Antibiofilm Peptides with MBIC Range 1–8 (μM) from the UniProt database

Name	Seq	Source	Predicted MBEC (μM)
sp—P0CF03—	FLGGLIGPLMSLIPGLLK	Ant	19.723
tr—A0A5K1B3V0—	VIRIGCKWKRTA	*Nymphaea colorata* (plant)	6.260
tr—A0A0E9SZ00—	MCTRWRVLLTCVRRR	*Anguilla anguilla* (eel)	28.711
tr—A0A2P2N8A3—	MGGKSDFRFCHVKKKVL	*Rhizophora mucronata* (plant)	11.138
tr—A0A0A9M1Q7—	MGRKFKWKLWT	*Arundo donax* (plant)	14.310
sp—C0HK43—	VNWKKILGKIIKVAK	*Lasioglossum laticeps* (bee)	3.071
tr—A0A0A9U210—	MTRIRRRRRHLLLLR	*Arundo donax* (plant)	22.612
sp—P17236—	FLPLILGKLVKGLL	Oriental hornet	36.241
tr—A0A2P2Q2Y8—	MLKLWLRIKLLRKAL	*Rhizophora mucronata* (plant)	35.601
tr—A0A5K1FWL9—	FRARLLRTAFR	*Nymphaea colorata* (plant)	22.722
sp—P82419—	GLVDVLGKVGGLIKKLLP	Ant	28.502
sp—C0HLD5—	FLSLIPKIAGGIASLVKNL	Frog	23.319
sp—P82420—	GLVDVLGKVGGLIKKLLPG	Ant	27.711
tr—E4Z311—	IKGILLRKIIKVR	*Oikopleura dioica* (tunicate)	35.514

Table S12. Newly Predicted Antibiofilm Peptides with MBIC Range 8–16 (μM) from the UniProt database

**Name**	**Seq**	**Source**	**Predicted MBEC (μM)**
tr—A0A3D5SU75—	PCPCGSGKKYKHCHGKLS	*Rhodocyclaceae bacterium*	1.854
sp—P0C424—	CCAPSACRLGCRPCCR	*Conus marmoreus* (marble cone)	2.968
tr—A0A5K1BN05—	LGCGHGLPGIFACLK	*Nymphaea colorata* (plant)	3.071
tr—A0A5K1DCQ4—	LGCGHGLPGIYACLK	*Nymphaea colorata* (plant)	3.071
sp—C0HK42—	VNWKKVLGKIIKVAK	*Lasioglossum laticeps* (bee)	3.071
sp—P30259—	GCKKRKARKRPKCKKARKRPKCKRRKVAKKKC	Catshark	4.290
sp—P14215—	RRWCFRVCYRGFCYRKCR	Atlantic horseshoe crab	6.030
sp—P14216—	RRWCFRVCYKGFCYRKCR	Atlantic horseshoe crab	6.030
tr—A0A0G3VIX2—	AVPSWRIKSWNR	Bacteria	7.090
tr—E9I8P2—	MLKKFLGKSGRRILR	*Solenopsis invicta* (ant)	8.156
sp—P69135—	KWCFRVCYRGICYRRCR	Asian horseshoe crab	18.171
sp—Q8WMD3—	MARYRRCRSRSRCRRRRRRCHRRRRRCCRRRRRRRA CCRRYRCRRR	Bat	21.567
tr—A0A0D3HK27—	MFGGSGPLKLL	*Oryza barthii* (plant)	22.009
tr—A0A1C8YA26—	FLGCRVQLAIKISGI	*Triticum aestivum* x *Aegilops sharonensis* (plant)	22.359
tr—A0A3Q7GQZ6—	GLAYRLVNLHFCKTKR	*Solanum lycopersicum* (tomato)	22.657
tr—A0A0K1NW40—	KAIALALGKSGCK	*Lycium cestroides* (plants)	22.679
sp—P0C022—	INLLKIAKGIIKSL	Wasp	22.679
sp—P85874—	INWKKIASIGKEVLKAL	Wasp	22.679
sp—P69034—	INWLKLGKKVSAIL	Wasp	22.679
sp—P69036—	INWLKLGKAVIDAL	Wasp	22.679
sp—P85443—	GLLDFLKAAGKGLVTNL	Frog	22.691
tr—A0A5K0UXG7—	ALLKSKPKLLRSGL	*Nymphaea colorata* (plant)	22.694
tr—E9JAR4—	KLVLRRILALCIIAVCK	Solenopsis invicta (ant)	22.924
sp—P82282—	IIGPVLGMVGSALGGLLKKIG	Toad	23.278
sp—P82285—	IIGPVLGLVGSALGGLLKKIG	Toad	23.278
sp—P85982—	IFGAILPLALGALKNLIK	Frog	23.712
LFB0040	FKCRRWAWRMKKLGA	Synthetic	25.902
sp—P0C1R0—	ILGTILGLLKSL	Wasp	32.702
tr—A0A5K0UVL7—	ILLIKVGCCKIK	*Nymphaea colorata* (plant)	38.404
LFB0017	FKCRRWQWR	Lactoferrin, source: cow	38.492
sp—C0HLM2—	SGCCKHPACGKNRC	Alpha-conotoxin, source: conus purpurascens	39.130
tr—A0A0K0LBU6—	GGYCGGAFRQRCICYRK	*Androctonus bicolor* (scorpion)	46.616
tr—A0A5K1F988—	EKFKIHKSGKRWM	*Nymphaea colorata* (plant)	46.812
tr—I7DAN2—	KKSGKIKSAYKR	*Rhea americana* (bird)	50.828
tr—S7IKV4—	SGLFCKGCSKL	*Chlamydia psittaci* (bacteria)	60.154
tr—Q16228—	WRWRRACRRPGRPFWRV	Human	61.163

Table S13. Newly Predicted Antibiofilm Peptides with MBIC Range 16–32 (μM) from the UniProt database

**Name**	**Seq**	**Source**	**Predicted MBEC (μM)**
sp—P0C005—	GLLKRIKTLL	Wasp	22.679
sp—P17238—	INLKAIAALVKKVL	Hornet	22.679
tr—Q9U8M9—	AGLGGIGLDTNREIVKSGPK	*Scaptomyza graminum* (insect)	20.318

7 DATASET

7.1 Positive Dataset

The details of our positive dataset, including the peptide sequence and its length, are given in Tables S14–S18.

Table S14. Peptide List for our Positive Dataset

Name	Seq	Seq Length
BREVININ-1GHA	FLGAVLKVAGKLVPAAICKISKKC	24
DERMASEPTIN-AC4	SLWGKLKEMAAAAGKAALNAVNGLVNQ	27
RPDEF1ALPHA	GFGCPNDYSCSNHCRDSIGCRGGYCKYHVICTCYGCKKRRSIQE	44
KASSINIATUERIN-3	FIQHLIPLIPHAIQGIKDIF	20
AGELAIA-MP	INWLKLGKAIIDAL	14
CCL20	SNFDCCLGYTDRILHPKFIVGFTRQLANEGCDINAIIFHTKKKLSVCANPKQTWVKYIVRLLS KKVKNM	69
CHICKEN	RFGRFLRKIRRFRPKVTITIQGSARFG	27
CITROPIN	GLFDVIKKVASVIGGL	16
COLISTIN	KTKKKLLKKT	10
CON10	FWSFLVKAASKILPSLIGGGDDNKSSS	27
COPRISIN	VTCDVLSFEAKGIAVNHSACALHCIALRKKGGSCQNGVCVCRN	43
DATUCIN	TFPKCAPTRPPGPKPCDINNFKSKFWHIWRA	31
DERMASEPTIN-PH	ALWKEVLKNAGKAALNEINNLV	22
DERMASEPTIN-PT9	GLWSKIKDAAKTAGKAALGFVNEMV	25
DHVAR4	KRLFKKLLFSLRKY	14
ENTEROCIN	LGSCVANKIKDEFFAMISISAIVKAAQKKAWKELAVTVLRFAKANGLKTNAIIVAGQLALWAV QCGLS	68
ESCULENTIN	GIFSKLAGKKIKNLLISGLKG	21
GL13K	GKIIKLKASLKLL	13
GRAMICIDIN	VKLFPVKLFP	10
HS02	KWAVRIIRKFIKGFIS	16
HUMAN defensin	GIINTLQKYYCRVRGGRCAVLSCLPKEEQIGKCSTRGRKCCRRKK	45
HYICIN	NKGCSACAIGAACLADGPIPDFEVAGITGTFGIAS	35
INDOLICIDIN	ILPWKWPWWPWRR	13
JAPONICIN-2LF	FIVPSIFLLKKAFCIALKKC	20
LL-37	LLGDFFRKSKEKIGKEFKRIVQRIKDFLRNLVPRTES	37
MP-C	LNLKALLAVAKKIL	14
MORONECIDIN-	FFRNLWKGAKAAFRAGHAAWRA	22
MYXINIDIN	GIHDILKYGKPS	12
NA-CATH	GLLSGILGAGKKIVF	15
NISIN	ITSISLCTPGCKTGALMGCNMKTATCHCSIHVSK	34
PARACENTRIN	EVASFDKSKLK	11
PHYLLOSEPTIN-1	FLSLIPHIVSGVASIAKHF	19
PHYLLOSEPTIN-CO	FLSMIPKIAGGIASLVKNL	19
PHYLLOSEPTIN-PHA	FLSLIPAAISAVSALANHF	19
PLEUROCIDIN	GWGSFFKKAAHVGKHVGKAALTHYL	25
POLYBIA-MP-II	INWLKLGKMVIDAL	14
POLYMYXIN	KTKKKFLKKT	10
PROTEGRIN	RGGRLCYCRRRFCVCVGR	18
SA-CATH	KFFKKLKKSVKKHVKKFFKKPKVIGVSIPF	30
SAAP-148	LKRVWKRVFKLLKRYWRQLKKPVR	24
SMAP-29-APD	RGLRRLGRKIAHGVKKYGPTVLRIIRIAG	29
TACHYPLESIN	KWCFRVCYRGICYRKCR	17
TEMPORIN-1OLA	FLPFLKSILGKIL	13

Table S15. Peptide List for our Positive Dataset (Cont.)

Name	Seq	Seq Length
TEMPORIN-B	LLPIVGNLLKSLL	13
TEMPORIN-1CEB	ILPILSLIGGLLGK	14
TEMPORIN-GHC	FLQHIIGALTHIF	13
TEMPORIN-GHD	FLQHIIGALSHFF	13
TEMPORIN-PTA	FFGSVLKLIPKIL	13
TETRAF2W-RK	WWWLRKIW	8
TOAP1	FIGMIPGLIGGLISAFK	17
TOAP2	FFGTLFKLGSKLIPGVMKLFSKKKER	26
TSAP-2	FLGMIPGLIGGLISAFK	17
UYCT3	ILSAIWSGIKSLF	13
VLL-28	VLLVTLTRLHQRGVIYRKWRHFSGRKYR	28
ZMD32	RTCQSQSHRFRGPCLRRSNCANVCRTEGFPGGRCRGFRRRCFCTTHC	47
BMAP-27	GRFKRFRKKFKKLFKKLSPVIPLLHL	26
BMAP-28	GGLRSLGRKILRAWKKYGPIIVPIIRI	27
SMAP-29	RGLRRLGRKIAHGVKKYGPTVLRIIRIA	28
KSL	KKVVFKVKFK	10
F2-5-12W	RWGRWLRKIRRWRPK	15
LL-31	LLGDFFRKSKEKIGKEFKRIVQRIKDFLRNL	31
LL7-31	RKSKEKIGKEFKRIVQRIKDFLRNL	25
LL13-37	IGKEFKRIVQRIKDFLRNLVPRTES	25
LL7-37	RKSKEKIGKEFKRIVQRIKDFLRNLVPRTES	31
LL-19	LLGDFFRKSKEKIGKEFKR	19
LL-25	LLGDFFRKSKEKIGKEFKRIVQRIK	25
LL-13	LLGDFFRKSKEKI	13
LL13-31	IGKEFKRIVQRIKDFLRNL	19
LL7-25	RKSKEKIGKEFKRIVQRIK	19
LL13-25	IGKEFKRIVQRIK	13
LL19-37	RIVQRIKDFLRNLVPRTES	19
KSL-W	KKVVFWVKFK	10
KS-30	KSKEKIGKEFKRIVQRIKDFLRNLVPRTES	30
KR-20	KRIVQRIKDFLRNLVPRTES	20
KR-12	KRIVQRIKDFLR	12
LACTOFERRICIN-(17-30)	FKCRRWQWRMKKLG	14
LACTOFERRAMPIN	WKLLSKAQEKFGKNKSR	17
MUC7-12-MER-L	RKSYKCLHKRCR	12
G10KHC	KKHRKHRKHRKHGGSGGSKNLRRIIRKGIHIIKKYG	36
MUC7-12-MER-L4	RKSYKALHKRAR	12
MUC7-20-MER	LAHQKPFIRKSYKCLHKRCR	20
HSN5	AKRHHGYKRKFH	12
MAGAININ-II	GIGKFLHSAKKFGKAFVGEIMNS	23
LYS-A1	KIFGAIWPLALGALKNLIK	19
AAP2	FHFFHHFFHFFHHF	14
CSP	SGSLSTFFRLFNRSFTQALGK	21
CSPC16	TFFRLFNRSFTQALGK	16
G2	KNLRIIRKGIHIIKKY	16
C16G2	TFFRLFNRSFTQALGKGGGKNLRIIRKGIHIIKKY	35
M8G2	TFFRLFNRGGGKNLRIIRKGIHIIKKY	27
S6L3-33	FKKFWKWFRRF	11
C16-33	TRRRLFNRSFTQALGKSGGGFKKFWKWFRRF	31
M8-33	TFFRLFNRSGGGFKKFWKWFRRF	23
CECROPIN-A-(1-7)-MELITTIN	KWKLFKKIGAVLKVL	15
HH15	KRFRIRVRVIRK	12

Table S16. Peptide List for our Positive Dataset (Cont.)

Name	Seq	Seq Length
BAC2A	RLARIVVIRVAR	12
1026	VQWRIRVRVIKK	12
1029	KQFRIRVRV	9
1036	VQFRIRVRIVIRK	13
1037	KRFRIRVRV	9
HH2	VQLRIRVAVIRA	12
1002	VQRWLIVWRIRK	12
1003	IVWKIKRWWVGR	12
1004	RFWKVRVKYIRF	12
1008	RIKWIVRFR	9
HH7	VRLRIRVAVRRA	12
1010	IRWRIRVWVRRI	12
1011	RRWVVWRIVQRR	12
1012	IFWRRIVIVKKF	12
1013	VRLRIRVA	8
1016	LRIRWIFKR	9
HH8	VRLRIRVAVIRK	12
1020	VRLRIRWWVLRK	12
HH10	KRFRIRVAVRRA	12
1035	KRWRWIVRNIRR	12
1031	WRWRVRVWR	9
IMB-2	TFFRLFNRGGGWGSFFKKAAHVGKL	25
BAC8C	RIWVIWRR	8
PTP-7	FLGALFKALSKLL	13
HOLOTHURIOIDIN-1	HLGHHALDHLLK	12
HOLOTHURIOIDIN-2	ASHLGHHALDHLLK	14
TN-AFP1	LMCTHPLDCSN	11
COPRISIN-BAAMP	VTCDVLSFEAKGIAVNH	17
HISTATIN	DSHAKRHHGYKRKFHEKHHSHRGY	24
HST	AKRHHGYKRKFHGGG	15
DF17-6K	KKKKKKAAFAAWAAFAA	17
DF21-10K	KKKKKKKKKKAAFAAWAAFAA	21
CWR11	CWFWKWWRRRRR	12
CHRYSOPHSIN-1	FFGWLIKGAIHAGKAIHGLIHRRRH	25
RK1	RWKRWWRRKK	10
RK2	RKKRWWRRKK	10
(IRIK)	IRIKIRIK	8
(IRVK)	IRVKIRVKIRVK	12
ALPHA-DEFENSIN-3	DCYCRIPACIAGERRYGTCIYQGRLWAFCC	30
BETA-DEFENSIN-1	DHYNCVSSGGQCLYSACPIFTKIQGTCYRGKAKCCK	36
MAGAININ-I	GIGKFLHSAGKFGKAFVGEIMKS	23
RIP	YSPWTNF	7
K4-S4(1â€“13)A	ALWKTLLKKVLKA	13
DD13-RIP	ALWKTLLKKVLKAYSPWTNF	20
2C-4	RWRWRWF	7
SM6(L1)2C	FIKHFIHRFGGGRWRWRWF	19
SM6(L3)2C	FIKHFIHRFSATRWRWRWF	19
SM6(L1)B33	FIKHFIHRFGGGFKKFWKWFRRF	23
NRC-16	GWKKWLRKGAKHLGQAAIK	19
GK7	GQIINLK	7
(RW)2-NH2	RWRW	4
(RW)3-NH2	RWRWRW	6

Table S17. Peptide List for our Positive Dataset (Cont.)

Name	Seq	Seq Length
(RW)4-NH2	RWRWRWRW	8
LASIO-III	VNWKKILGKIIKVVK	15
MELITTIN	GIGAVLKVLTTGLPALISWIKRKRQQ	26
MELIMINE	TLISWIKNKRKQRPRVSRRRRRRGGRRRR	29
MELIMINE-CYSN	CTLISWIKNKRKQRPRVSRRRRRRGGRRRR	30
MELIMINE-CYSC	TLISWIKNKRKQRPRVSRRRRRRGGRRRRC	30
MELIMINE-CYS13	TLISWIKNKRKQCRPRVSRRRRRRGGRRRR	30
K4-S4(1-15)A	LWKTLLKKVLKAAA	14
BETA6-20-G3K6	NEEGFFSARGHRPLDGGGKKKKKK	24
HEPCIDIN	ICIFCCGCCHRSKCGMCCKT	20
NA-CATH-BAAMP	KRFKKFFKKLKNSVKKRAKKFFKKPKVIGVTFPF	34
NA-CATH-ATRA1-ATRA1	KRFKKFFKKLKNSVKKRFKKFFKKLKVIGVTFPF	34
LACTOFERRICIN-B-(17-41)	FKCRRWQWRMKKLGAPSITCVRRAF	25
SCRAMBLED	GLKLRFEFSKIKGEFLKTPEVRFRDIKLKDNRISVQR	37
R-FV-I16	RFRRLFRIRVRVLKKI	16
FV7	FRIRVRV	7
VSL2	AFKAFWKFVKFVK	13
VS2	KWFWKFVKFVK	11
L-K6	IKKILSKIKKLLK	13
HLF1-11	GRRRRSVQWCA	11
FS3	YAPWTNF	7
TET-213	KRWWKWWRRC	10
1010CYS	IRWRIRVWVRRIC	13
TET-20	KRWRIRVRVIRKC	13
TET-26	WIVVIWRRKRRRC	13
FS8	YAPWTNA	7
CHROMOFUNGIN	RILSILRHQNLLKELQDLAL	20
CECROPIN-B	KWKVFKKIEKMGRNIRNGIVKAGPAIAVLGEAKAL	35
MAGAININ	GIGLFLHSAGLFGLAFVGEIMKS	23
CYSLASIO-III	CVNWKKILGKIIKVVK	16
DASAMP1	FFGKVLKLIRKIF	13
BMAP-18	GRWKRWRKKWKKLWKKLS	18
BACTENECIN	RLCRIVVIRVCR	12
CA-MA	KWKLFKKIGIGKFLHSAKKF	20
RTA3	RPAFRKAAFRVMRACV	16
DHVAR5	LLLFLLKKRKKRKY	14
KABT-AMP	GIWKKWIKKWLKKLLKKLWKKG	22
P10	LAREYKKIVEKLKRWLRQVLRTLR	24
P60.4AC	IGKEFKRIVERIKRFLRELVRPLR	24
OSIP108	MLCVLQGLRE	10
S-OSIP108	ELRLVCMGQL	10
[CYC2]OSIP108	MLCVLQGLREGG	12
[CYC3]OSIP108	MLCVLQGLREC	11
1018	VRLIVAVRIWRR	12
HE1	RRWIRVAVILRV	12
HE2	VRLIRAVRAWRV	12
HE3	VRWARVARILRV	12
HE4	VRLIWAVRIWRR	12
HE10	VRLIVRIWRR	10
HE12	RFKRVARVIW	10
GL13KR1	IGIKLLKSKLKAL	13
(IKIK)2	IKIKIKIK	8

Table S18. Peptide List for our Positive Dataset (Cont.)

Name	Seq	Seq Length
RI1012	FKKVIVIRRWFI	12
RI1002	KRIRWVILWRQV	12
LJK1	VFLRRIRVIVIR	12
RIJK1	RIVIVRIRRLFV	12
LJK2	VFWRRIRVWVIR	12
RIJK2	RIVWVRIRRWFV	12
LJK3	VQLRAIRVRVIR	12
RIJK3	RIVRVRIARLQV	12
LJK4	VQLRRIRVWVIR	12
RIJK4	RIVWVRIRRLQV	12
LJK5	VQWRAIRVRVIR	12
RIJK5	RIVRVRAIRWQV	12
LJK6	VQWRRIRVWVIR	12
RIJK6	RIVWVRIRRWQV	12
NAL-P-113	AKRRRGYKRKFKK	13
P15	GTPGPQGIAGQRGVV	15
P15-CSP	GTPGPQGIAGQRGVVAEAAAKEAAAKEAAAKASGSLSTFFRLFNRSFTQALGK	53
C-GG-NT-DHVAR5	CGGLLLFLLKKRKKRKY	17
KT2	NGVQPKYKWWKWWKKWW	17
RT2	NGVQPKYRWWRWWRRWW	17
LF11-322	PFWRIRIRR	9
LF11-324	PFFWRIRIRR	10
6-MO-LF11-227	FWRRFWRR	8
LF11-215	FWRIRIRR	8
D-ATRA-1A	KRAKKFFKKLK	11
ATRA-2	KRAKKFFKKPK	11
ATRA-1	KRFKKFFKKLK	11
ALL	LKLLKKLLKKLLKLL	15
SEG5D	KKKLLLLLLLLLKKK	15
SEG6D	LLLLLKKKKKKLLLL	15
G10	KNLRRIIRKGIHIIKKYG	18
LIN-SB056	WKKIRVRLSA	10
LIN-SB056-1	KWKIRVRLSA	10
MYXINIDIN2	KIKWILKYWKWS	12
MYXINIDIN3	RIRWILRYWRWS	12
GH12	GLLWHLLHHLLH	12
PA-MAP	LAAKLTKAATKLTAALTKLAAALT	24
HSAFP1	DGVKLCDVPSGTWSGHCGSSSKCSQQCKDREHFAYGGACHYQFPSVKCFCKRQC	54
HSLIN06	EHFAYGGAKHYQFPSVKKFKKRQK	24
Verine	RRRWWWWV	8
Phylloseptin-PTa	FLSLIPKIAGGIAALAKHL	19

7.2 MBEC Dataset

Antibiofilm peptides with MBEC values are listed in Tables S19–S20. The pathogens against which the MBEC values are effective are also listed in the 'pathogen' column. The MBEC values are listed in μM.

Table S19. Antibiofilm Peptides and MBEC (μM) Values

Name	Seq	MBEC (μM)	Pathogen	Source
BREVININ-1GHA	FLGAVLKVAGKLVPAAICKISKKC	16	*S. aureus*	Chen et al. (2018)
DERMASEPTIN-AC4	SLWGKLKEMAAAAGKAALNAVNGLVNQ	256	*S. aureus*	Gong et al. (2020)
KASSINIATUERIN-3	FIQHLIPLIPHAIQGIKDIF	64	*S. aureus*	Wang et al. (2020)
CCL20	SNFDCCLGYTDRILHPKFIVGFTRQLANEGCDINAIIFH TKKKLSVCANPKQTWVKYIVRLLSKKVKNM	128	*P. aeruginosa*	Ramamourthy et al. (2019)
COPRISIN	VTCDVLSFEAKGIAVNHSACALHCIALRKKGGSCQNG VCVCRN	4.49	*P. aeruginosa*	Hwang et al. (2013)
DERMASEPTIN-PH	ALWKEVLKNAGKAALNEINNLV	128	*S. aureus*	Huang et al. (2017)
DERMASEPTIN-PT9	GLWSKIKDAAKTAGKAALGFVNEMV	32	*S. aureus*	Li et al. (2019)
ESCULENTIN	GIFSKLAGKKIKNLLISGLKG	6	*P. aeruginosa*	Luca et al. (2013)
GL13K	GKIIKLKASLKLL	22.47	*P. aeruginosa*	Hirt and Gorr (2013)
HUMAN defensin	GIINTLQKYYCRVRGGRCAVLSCLPKEEQIGKCSTRGR KCCRRKK	3.1	*MRSE*	Sutton and Pritts (2014)
INDOLICIDIN	ILPWKWPWWPWRR	335.7	*MRSA*	Mataraci and Dosler (2012)
LL-37	LLGDFFRKSKEKIGKEFKRIVQRIKDFLRNLVPRTES	20	*P. aeruginosa*	Nagant et al. (2012)
NISIN	ITSISLCTPGCKTGALMGCNMKTATCHCSIHVSK	183.1	*MRSA*	Mataraci and Dosler (2012)
PHYLLOSEPTIN-1	FLSLIPHIVSGVASIAKHF	5	*S. aureus*	Zhang et al. (2010)
PLEUROCIDIN	GWGSFFKKAAHVGKHVGKAALTHYL	23.62	*S. mutans*	Tao et al. (2011)
BMAP-27	GRFKRFRKKFKKLFKKLSPVIPLLHL	6.2	*P. aeruginosa*	Pompilio et al. (2011)
BMAP-28	GGLRSLGRKILRAWKKYGPIIVPIIRI	6.5	*P. aeruginosa*	Pompilio et al. (2011)
SMAP-29	RGLRRLGRKIAHGVKKYGPTVLRIIRIA	6.25	*P. aeruginosa*	Pompilio et al. (2011)
KSL	KKVVFKVKFK	400	*S. mutans*	Liu et al. (2011)
F2-5-12W	RWGRWLRKIRRWRPK	40	*S.epidermidis*	Molhoek et al. (2011)
LL-31	LLGDFFRKSKEKIGKEFKRIVQRIKDFLRNL	20	*P. aeruginosa*	Nagant et al. (2012)
LL13-37	IGKEFKRIVQRIKDFLRNLVPRTES	100	*P. aeruginosa*	Nagant et al. (2012)
LL7-37	RKSKEKIGKEFKRIVQRIKDFLRNLVPRTES	50	*P. aeruginosa*	Nagant et al. (2012)
KSL-W	KKVVFWVKFK	191.16	*MRSA*	Gawande et al. (2014)
MUC7-12-MER-L	RKSYKCLHKRCR	12.5→50; median 50	*S. mutans*	Wei et al. (2006)
MUC7-12-MER-L4	RKSYKALHKRAR	50; median >50	*S. mutans*	Wei et al. (2006)
MUC7-20-MER	LAHQKPFIRKSYKCLHKRCR	6.25–25; median 25	*S. mutans*	Wei et al. (2006)
HSN5	AKRHHGYKRKFH	12.5→50; median >50	*S. mutans*	Wei et al. (2006)
MAGAININ-II	GIGKFLHSAKKFGKAFVGEIMNS	25→50; median >50	*S. mutans*	Wei et al. (2006)
C16G2	TFFRLFNRSFTQALGKGGGKNLRIIRKGIHIIKKY	25	*S. mutans*	Sullivan et al. (2011)
BAC8C	RIWVIWRR	108.13	*S. mutans*	Ding et al. (2014)
PTP-7	FLGALFKALSKLL	40	*S. aureus*	Kharidia and Liang (2011)
CHRYSOPHSIN-1	FFGWLIKGAIHAGKAIHGLIHRRRH	11.07	*S. aureus*	Wang et al. (2012)
2C-4	RWRWRWF	50	*S. mutans and other oral streptococci*	He et al. (2010)
SM6(L1)2C	FIKHFIHRFGGGRWRWRWF	50	*S. mutans and other oral streptococci*	He et al. (2010)
SM6(L3)2C	FIKHFIHRFSATRWRWRWF	50	*S. mutans and other oral streptococci*	He et al. (2010)
SM6(L1)B33	FIKHFIHRFGGGFKKFWKWFRRF	50	*S. mutans*	He et al. (2010)
(RW)4-NH2	RWRWRWRW	100	*E.coli*	Hou et al. (2010)
MELITTIN	GIGAVLKVLTTGLPALISWIKRKRQQ	50	*S.mutans*	Sullivan et al. (2011)
R-FV-I16	RFRRLFRIRVRVLKKI	64	*P. aeruginosa*	Xu et al. (2014)
L-K6	IKKILSKIKKLLK	6.25	*S. mutans*	Shang et al. (2014)
CA-MA	KWKLFKKIGIGKFLHSAKKF	361.54	*MRSA*	Mataraci and Dosler (2012)
P10	LAREYKKIVEKLKRWLRQVLRTLR	32	*MRSA*	Haisma et al. (2014)

Table S20. Antibiofilm Peptides and MBEC (μM) Values (Cont...)

Name	Seq	MBEC (μM)	Pathogen	Source
1018	VRLIVAVRIWRR	6.51	*P. aeruginosa*	de la Fuente-Núñez et al. (2013)
HE4	VRLIWAVRIWRR	6.16	*P. aeruginosa*	de la Fuente-Núñez et al. (2014)
HE10	VRLIVRIWRR	7.32	*P. aeruginosa*	de la Fuente-Núñez et al. (2014)
DJK5	VQWRAIRVRVIR	1.61	*P. aeruginosa*	de la Fuente-Núñez et al. (2015)
DJK6	VQWRRIRVWVIR	1.5	*P. aeruginosa*	de la Fuente-Núñez et al. (2015)
KT2	NGVQPKYKWWKWWKKWW	1	*E. coli*	Anunthawan et al. (2015)
RT2	NGVQPKYRWWRWWRRWW	1	*E. coli*	Anunthawan et al. (2015)
DI-MB-LF11-322	PFWRIRIRR	246.38	*P. aeruginosa*	Sánchez-Gómez et al. (2015)
LF11-324	PFFWRIRIRR	55.33	*P. aeruginosa*	Sánchez-Gómez et al. (2015)
6-MO-LF11-227	FWRRFWRR	489.01	*P. aeruginosa*	Sánchez-Gómez et al. (2015)
LF11-215	FWRIRIRR	133.41	*P. aeruginosa*	Sánchez-Gómez et al. (2015)
P60.4AC	IGKEFKRIVERIKRFLRELVRPLR	32	*MRSA*	Haisma et al. (2014)

REFERENCES

Anunthawan, T., de la Fuente-Núñez, C., Hancock, R. E., and Klaynongsruang, S. (2015). Cationic amphipathic peptides kt2 and rt2 are taken up into bacterial cells and kill planktonic and biofilm bacteria. *Biochimica et Biophysica Acta (BBA) - Biomembranes* 1848, 1352–1358. doi:https://doi.org/10.1016/j.bbamem.2015.02.021

[Dataset] Armstrong, D. and Zidovetzki, R. (2009). Helical wheel projections. `www.donarmstrong.com/cgi-bin/wheel.pl`. Version ID: wheel.pl,v 1.4 2009-10-20 21:23:36 don Exp

Chen, Q., Cheng, P., Ma, C., Xi, X., Wang, L., Zhou, M., et al. (2018). Evaluating the bioactivity of a novel broad-spectrum antimicrobial peptide brevinin-1gha from the frog skin secretion of hylarana guentheri and its analogues. *Toxins* 10. doi:10.3390/toxins10100413

de la Fuente-Núñez, C., Mansour, S. C., Wang, Z., Jiang, L., Breidenstein, E. B., Elliott, M., et al. (2014). Anti-biofilm and immunomodulatory activities of peptides that inhibit biofilms formed by pathogens isolated from cystic fibrosis patients. *Antibiotics* 3, 509–526. doi:10.3390/antibiotics3040509

de la Fuente-Núñez, C., Reffuveille, F., Mansour, S. C., Reckseidler-Zenteno, S. L., Hernández, D., Brackman, G., et al. (2015). D-enantiomeric peptides that eradicate wild-type and multidrug-resistant biofilms and protect against lethal Pseudomonas aeruginosa infections. *Chem Biol* 22, 196–205

de la Fuente-Núñez, C., Reffuveille, F., nde, L. F., and Hancock, R. E. (2013). Bacterial biofilm development as a multicellular adaptation: antibiotic resistance and new therapeutic strategies. *Current Opinion in Microbiology* 16, 580–589. doi:10.1016/j.mib.2013.06.013. Antimicrobials · Genomics

Ding, Y., Wang, W., Fan, M., Tong, Z., Kuang, R., Jiang, W., et al. (2014). Antimicrobial and anti-biofilm effect of Bac8c on major bacteria associated with dental caries and Streptococcus mutans biofilms. *Peptides* 52, 61–67

Gawande, P. V., Leung, K. P., and Madhyastha, S. (2014). Antibiofilm and antimicrobial efficacy of dispersinb®-ksl-w peptide-based wound gel against chronic wound infection associated bacteria. *Current Microbiology* 68, 635–641. doi:10.1007/s00284-014-0519-6

Gong, Z., Pei, X., Ren, S., Chen, X., Wang, L., Ma, C., et al. (2020). Identification and rational design of a novel antibacterial peptide dermaseptin-ac from the skin secretion of the red-eyed tree frog agalychnis callidryas. *Antibiotics* 9. doi:10.3390/antibiotics9050243

Gupta, S., Sharma, A. K., Jaiswal, S. K., and Sharma, V. K. (2016). Prediction of biofilm inhibiting peptides: An in silico approach. *Frontiers in Microbiology* 7, 949. doi:10.3389/fmicb.2016.00949

Haisma, E. M., de Breij, A., Chan, H., van Dissel, J. T., Drijfhout, J. W., Hiemstra, P. S., et al. (2014). Ll-37-derived peptides eradicate multidrug-resistant staphylococcus aureus from thermally wounded human skin equivalents. *Antimicrobial Agents and Chemotherapy* 58, 4411–4419. doi:10.1128/AAC.02554-14

He, J., Yarbrough, D. K., Kreth, J., Anderson, M. H., Shi, W., and Eckert, R. (2010). Systematic approach to optimizing specifically targeted antimicrobial peptides against ¡i¿streptococcus mutans¡/i¿. *Antimicrobial Agents and Chemotherapy* 54, 2143–2151. doi:10.1128/AAC.01391-09

Hirt, H. and Gorr, S.-U. (2013). Antimicrobial peptide gl13k is effective in reducing biofilms of pseudomonas aeruginosa. *Antimicrobial Agents and Chemotherapy* 57, 4903–4910. doi:10.1128/AAC.00311-13

Hou, S., Liu, Z., Young, A. W., Mark, S. L., Kallenbach, N. R., and Ren, D. (2010). Effects of trp- and arg-containing antimicrobial-peptide structure on inhibition of ¡i¿escherichia coli¡/i¿ planktonic growth and biofilm formation. *Applied and Environmental Microbiology* 76, 1967–1974. doi:10.1128/AEM.02321-09

Huang, L., Chen, D., Wang, L., Lin, C., Ma, C., Xi, X., et al. (2017). Dermaseptin-ph: A novel peptide with antimicrobial and anticancer activities from the skin secretion of the south american orange-legged leaf frog, pithecopus (phyllomedusa) hypochondrialis. *Molecules* 22. doi:10.3390/molecules22101805

Hwang, I.-s., Hwang, J.-S., Hwang, J. H., Choi, H., Lee, E., Kim, Y., et al. (2013). Synergistic effect and antibiofilm activity between the antimicrobial peptide coprisin and conventional antibiotics against opportunistic bacteria. *Current Microbiology* 66, 56–60. doi:10.1007/s00284-012-0239-8

Kharidia, R. and Liang, J. F. (2011). The activity of a small lytic peptide PTP-7 on Staphylococcus aureus biofilms. *J Microbiol* 49, 663–668

Li, M., Xi, X., Ma, C., Chen, X., Zhou, M., Burrows, J. F., et al. (2019). A novel dermaseptin isolated from the skin secretion of phyllomedusa tarsius and its cationicity-enhanced analogue exhibiting effective antimicrobial and anti-proliferative activities. *Biomolecules* 9. doi:10.3390/biom9100628

Liu, Y., Wang, L., Zhou, X., Hu, S., Zhang, S., and Wu, H. (2011). Effect of the antimicrobial decapeptide KSL on the growth of oral pathogens and Streptococcus mutans biofilm. *Int J Antimicrob Agents* 37, 33–38

Luca, V., Stringaro, A., Colone, M., Pini, A., and Mangoni, M. L. (2013). Esculentin(1-21), an amphibian skin membrane-active peptide with potent activity on both planktonic and biofilm cells of the bacterial pathogen pseudomonas aeruginosa. *Cellular and Molecular Life Sciences* 70, 2773–2786. doi:10.1007/s00018-013-1291-7

Madeira, F., Park, Y. M., Lee, J., Buso, N., Gur, T., Madhusoodanan, N., et al. (2019). The embl-ebi search and sequence analysis tools apis in 2019. *Nucleic acids research* 47, W636—W641. doi:10.1093/nar/gkz268

Mataraci, E. and Dosler, S. (2012). In vitro activities of antibiotics and antimicrobial cationic peptides alone and in combination against methicillin-resistant Staphylococcus aureus biofilms. *Antimicrob Agents Chemother* 56, 6366–6371

Molhoek, E. M., van Dijk, A., Veldhuizen, E. J., Haagsman, H. P., and Bikker, F. J. (2011). A cathelicidin-2-derived peptide effectively impairs Staphylococcus epidermidis biofilms. *Int J Antimicrob Agents* 37, 476–479

Nagant, C., Pitts, B., Nazmi, K., Vandenbranden, M., Bolscher, J. G., Stewart, P. S., et al. (2012). Identification of peptides derived from the human antimicrobial peptide ll-37 active against biofilms formed by pseudomonas aeruginosa using a library of truncated fragments. *Antimicrobial Agents and Chemotherapy* 56, 5698–5708. doi:10.1128/AAC.00918-12

Pompilio, A., Scocchi, M., Pomponio, S., Guida, F., Di Primio, A., Fiscarelli, E., et al. (2011). Antibacterial and anti-biofilm effects of cathelicidin peptides against pathogens isolated from cystic fibrosis patients. *Peptides* 32, 1807–1814

Ramamourthy, G., Arias, M., Nguyen, L. T., Ishida, H., and Vogel, H. J. (2019). Expression and purification of chemokine mip-3α (ccl20) through a calmodulin-fusion protein system. *Microorganisms* 7. doi:10.3390/microorganisms7010008

Sánchez-Gómez, S., Ferrer-Espada, R., Stewart, P. S., Pitts, B., Lohner, K., and Martínez de Tejada, G. (2015). Antimicrobial activity of synthetic cationic peptides and lipopeptides derived from human lactoferricin against pseudomonas aeruginosa planktonic cultures and biofilms. *BMC Microbiology* 15, 137. doi:10.1186/s12866-015-0473-x

Schiffer, M. and Edmundson, A. B. (1967). Use of helical wheels to represent the structures of proteins and to identify segments with helical potential. *Biophysical journal* 7, 121–135. doi:10.1016/S0006-3495(67) 86579-2

Shang, D., Liang, H., Wei, S., Yan, X., Yang, Q., and Sun, Y. (2014). Effects of antimicrobial peptide L-K6, a temporin-1CEb analog on oral pathogen growth, Streptococcus mutans biofilm formation, and anti-inflammatory activity. *Appl Microbiol Biotechnol* 98, 8685–8695

Singh, H., Singh, S., and Singh Raghava, G. P. (2019). Peptide secondary structure prediction using evolutionary information. *bioRxiv* doi:10.1101/558791

Sullivan, R., Santarpia, P., Lavender, S., Gittins, E., Liu, Z., Anderson, M. H., et al. (2011). Clinical efficacy of a specifically targeted antimicrobial peptide mouth rinse: Targeted elimination of ¡i¿streptococcus mutans¡/i¿ and prevention of demineralization. *Caries Research* 45, 415–428. doi:10.1159/000330510

Sutton, J. M. and Pritts, T. A. (2014). Human beta-defensin 3: a novel inhibitor of Staphylococcus-produced biofilm production. Commentary on "Human β-defensin 3 inhibits antibiotic-resistant Staphylococcus biofilm formation". *J Surg Res* 186, 99–100

Tao, R., Tong, Z., Lin, Y., Xue, Y., Wang, W., Kuang, R., et al. (2011). Antimicrobial and antibiofilm activity of pleurocidin against cariogenic microorganisms. *Peptides* 32, 1748–1754

Wang, H., He, H., Chen, X., Zhou, M., Wei, M., Xi, X., et al. (2020). A novel antimicrobial peptide (kassinatuerin-3) isolated from the skin secretion of the african frog, kassina senegalensis. *Biology (Basel)* 9

Wang, W., Tao, R., Tong, Z., Ding, Y., Kuang, R., Zhai, S., et al. (2012). Effect of a novel antimicrobial peptide chrysophsin-1 on oral pathogens and Streptococcus mutans biofilms. *Peptides* 33, 212–219

Waterhouse, A. M., Procter, J. B., Martin, D. M. A., Clamp, M., and Barton, G. J. (2009). Jalview version 2—a multiple sequence alignment editor and analysis workbench. *Bioinformatics* 25, 1189–1191. doi:10.1093/bioinformatics/btp033

Wei, G.-X., Campagna, A. N., and Bobek, L. A. (2006). Effect of muc7 peptides on the growth of bacteria and on streptococcus mutans biofilm. *Journal of Antimicrobial Chemotherapy* 57, 1100–1109. doi:10.1093/jac/dkl120

Xu, W., Zhu, X., Tan, T., Li, W., and Shan, A. (2014). Design of embedded-hybrid antimicrobial peptides with enhanced cell selectivity and anti-biofilm activity. *PLOS ONE* 9, 1–13. doi:10.1371/journal.pone.0098935

Zhang, R., Zhou, M., Wang, L., McGrath, S., Chen, T., Chen, X., et al. (2010). Phylloseptin-1 (psn-1) from phyllomedusa sauvagei skin secretion: a novel broad-spectrum antimicrobial peptide with antibiofilm activity. *Molecular immunology* 47 11-12, 2030–7
